# Discrepancies in the rumen microbiome, metabolome, and serum metabolome among Hu sheep, East Friesian sheep, and East Friesian × Hu crossbred sheep

**DOI:** 10.3389/fmicb.2025.1498050

**Published:** 2025-04-28

**Authors:** Ruilin Du, Shuo Yan, Wenna Yao, Huimin Zhang, Yue Xue, Yulong Zhao, Guifang Cao, Jun Liu, Yong Zhang, Xihe Li, Siqin Bao, Yongli Song

**Affiliations:** ^1^Research Center for Animal Genetic Resources of Mongolia Plateau, College of Life Sciences, Inner Mongolia University, Hohhot, China; ^2^The State Key Laboratory of Reproductive Regulation and Breeding of Grassland Livestock, College of Life Sciences, Inner Mongolia University, Hohhot, China; ^3^Key Laboratory of Animal Biotechnology of the Ministry of Agriculture, College of Veterinary Medicine, Northwest A&F University, Yangling, China; ^4^Inner Mongolia Saikexing Institute of Breeding and Reproductive Biotechnology in Domestic Animal, Hohhot, China

**Keywords:** sheep, crossbred sheep, rumen microbiome, untargeted metabolomics, bile acids

## Abstract

Crossbreeding has emerged as a strategy to combine desirable traits from different sheep breeds, with the goal of enhancing productivity, disease resistance, and growth rates. This study compares the immune responses, rumen microbiomes, and serum metabolites of Hu sheep, East Friesian (EF) sheep, and crossbred Hu × EF (DH) sheep to explore the effects of crossbreeding on productivity and disease resistance. Hu sheep exhibited significantly higher lymphocyte counts (*p* < 0.05) and white blood cell (WBC) counts (*p* < 0.05) compared to EF and DH sheep, indicating stronger basal immune responses. DH sheep showed superior immune responses, with a higher cluster of differentiation 4+/cluster of differentiation 8+ (CD4+/CD8+) T cell ratio (*p* < 0.05) compared to EF sheep. Rumen microbiome analysis revealed distinct microbial profiles; DH sheep exhibited higher relative abundances of Prevotella (*p* < 0.05), which is associated with improved growth and disease resistance. Metabolomic analysis revealed significant differences in bile acid profiles: DH sheep exhibited higher levels of 6-keto lithocholic acid (6-ketoLCA), cholic acid and chenodeoxycholic acid (CDCA), and 3*β*-hyodeoxycholic acid (3β-HDCA) (*p* < 0.05), which is associated with improved immune function and gut health. These results indicate that crossbreeding improves immune resilience and metabolic efficiency, which has implications for breeding strategies designed to enhance livestock productivity and disease resistance.

## 1 Background

Mutton is known for being a high-quality meat rich in essential polyunsaturated fatty acids, minerals, and vitamins (Ponnampalam et al., 2009), and global consumer demand for both mutton and sheep’s milk is on the rise (de Andrade et al., 2016). This has resulted in an increased focus on boosting sheep productivity, with crossbreeding emerging as a key strategy to combine beneficial traits from different breeds, thereby improving growth, feed efficiency, and disease resistance (Roschinsky et al., 2015; Battacone et al., 2021). In China, although native breeds such as Hu sheep exhibit strong disease resistance and environmental adaptability, they show lower growth rates and meat yields compared to foreign breeds such as East Friesian (EF) sheep, which are known for their high milk production and rapid growth (Luo et al., 2022; Li et al., 2021; Feng et al., 2018; Wang et al., 2020). Crossbreeding dairy and mutton breeds, such as Hu and EF sheep, is promising for improving lamb productivity and disease resistance (Lu et al., 2022; Ellies-Oury et al., 2022).

Hu sheep demonstrate strong environmental adaptability, thriving in hot and humid conditions as well as, more recently, in arid and cold northern regions, exhibiting robust production performance (Xu et al., 2022). East Friesian (EF) sheep, native to East Frisia, Germany, are recognized as a dual-purpose breed known for their large body size, high milk production, and rapid growth rates. However, they have relatively low disease resistance compared to Hu sheep (Angeles-Hernandez et al., 2017). EF sheep are ideal candidates for crossbreeding in meat sheep production due to their high reproductive efficiency, milk yield, and growth rate (Yan et al., 2024; Nguyen et al., 2018; Kominakis et al., 2017). Crossbreeding EF sheep with native breeds aims to enhance not only production and meat yield but also disease resistance (Jiang et al., 2019; Russell et al., 1992).

The rumen microbiome, consisting of diverse microbial communities, plays a crucial role in digestion, nutrient absorption, and energy production in ruminants (Mizrahi et al., 2021; Mizrahi and Jami, 2018; Matthews et al., 2019; Thomas et al., 2017). It also impacts feed efficiency and methane emissions (Friedman et al., 2017; O'Hara et al., 2020; Furman et al., 2020), making it an important factor in livestock production. Recent studies indicate that the rumen microbiome can vary significantly between breeds, influencing traits such as growth, milk production, and feed conversion (Difford et al., 2018; Sasson et al., 2017; Xie et al., 2021; Li et al., 2019; Bickhart and Weimer, 2018; Myer et al., 2015). However, little is known about how crossbreeding affects the microbiome, particularly in terms of methanogenic bacteria and methane emissions, as well as how the rumen microbiome varies between Hu sheep, EF sheep, and their crossbreeds.

This study aims to address this gap by examining the rumen microbiome composition in Hu, EF, and crossbred sheep. Understanding these differences may yield insights into optimizing breeding strategies for improved productivity and environmental sustainability.

## 2 Methods

### 2.1 The sheep sample collection

In this study, we used six healthy male Hu sheep (2 months old), six healthy male East Friesian × Hu F1 crossbred sheep (2 months old), and six healthy male East Friesian sheep (2 months old). The Hu sheep and East Friesian × Hu F1 crossbred sheep were purchased from the Inner Mongolia Shengle Biotechnology Company (Hohhot, Inner Mongolia, China), while the East Friesian sheep were purchased from the Leke Biotechnology Company (Hohhot, Inner Mongolia, China). All sheep were housed at the experimental farm of Shengle Biotechnology for over a month, fed hay and extender, and had *ad libitum* access to water and minerals; their weight ranged 15 ± 3 kg, and they were kept under identical conditions.

The sheep were anesthetized with intravenous thiopental (0.125 mg/kg, Kangjiano Biological Co., Ltd., Suzhou, China) and euthanized with intravenous potassium chloride (5–10 mL, Sinopharm Chemical Reagent Co., Ltd., Shanghai, China), following standard protocols (Dias et al., 2018). We collected rumen digesta and blood samples from each breed (*n* = 6), resulting in a total of 18 samples. Rumen tissue samples were washed with PBS, and rumen contents were taken immediately after opening the rumen using sterile medical gauze to prevent contamination. Blood was collected from the jugular vein, and 5 mL was placed in ethylenediaminetetraacetic acid (EDTA) tubes and stored at −80°C. The serum was separated by centrifugation (1,300 *g*, 15 min, 4°C) into three aliquots: two were frozen at −20°C for inflammatory cytokine assays, and one was stored at −80°C for serum metabolome analysis. Rumen digesta samples were stored in 2.5 mL tubes at −80°C for microbiome and metabolite analysis. All fresh samples were frozen in liquid nitrogen for above 30 min before being stored at −80°C for DNA isolation.

### 2.2 Determination of immunoglobulin a (IgA), IgM, IgG, CD4, and CD8 levels in serum

The IgA, IgM, IgG, CD4, and CD8 enzyme-linked immunosorbent assay (ELISA) kits were applied to examine the levels of IgA, IgM, IgG, CD4, and CD8 in serum on the basis of the manufacturer’s instructions. IgA ELISA kits (YX-090701S), IgM ELISA kits (YX-090713S), IgG ELISA kits (YX-090707S), CD4 ELISA kits (YX-030404S), and CD8 ELISA kits (YX-030408) were purchased from He Peng, Biotechnology Co., Ltd. (Shanghai, China).

## 3 Microbiome and metabolites analysis

### 3.1 16S ribosomal RNA (rRNA) gene and metagenomic sequencing

16S rRNA gene and metagenomic sequencing of rumen digesta samples (*n* = 6 for each sheep breed) were performed by Wuhan Metware Metabolic Biotechnology Co., Ltd. (Wuhan, Hubei, China). The results were analyzed using the Metware Cloud platform.

### 3.2 Microbiome analysis

#### 3.2.1 Sequencing data processing

The data for each sample were split based on the barcode and polymerase chain reaction (PCR) primer sequences, with barcode and primer sequences compared as per previous methods (Edgar, 2013). The original reads were filtered using fastp (Shenzhen, China) (v0.22.0),^1^ applying the following criteria: removal of joint sequences, exclusion of reads with 15 or more N bases, removal of reads with >50% low-quality bases (mass value ≤ 20), deletion of reads with an average mass < 20 in a 4-base window, removal of polyG tails, and elimination of reads <150 bp. The high-quality paired-end reads were merged using FLASH (Cambridge, United States) (v1.2.11),^2^ generating clean tag data. These tags were processed with vsearch (Oslo, Norway) (v2.22.1) for chimera detection, using a species annotation database,^3^ and the chimeric sequences were removed to obtain the final effective tags.

#### 3.2.2 *α*-diversity analysis

The α-diversity indices [Chao1, Shannon, Simpson, albumin, C-reactive protein, and endoscopy (ACE)] were calculated using the photoseq (Seattle, United States) (v1.40.0) and vegan (Oulu, Finland) (v2.6.2) packages in R (Vienna, Austria) (v4.2.0). The dilution curves, rank abundance curves, and species accumulation curves were also plotted in R. The intergroup differences in α-diversity were analyzed using both parametric and nonparametric tests. One-way analysis of variance (ANOVA) and Student’s *t*-test were performed using Statistical Package for the Social Sciences (SPSS v22.0, IBM, Chicago, IL, USA).

#### 3.2.3 *β*-diversity analysis

The diversity analysis was performed using the photoseq (Seattle, United States) (v1.40.0) package in R (v4.2.0) to calculate the unique fraction (UniFrac) distance and construct an unweighted pair group method with arithmetic mean (UPGMA) clustering tree. R was used to generate principal component analysis (PCA), principal coordinate analysis (PCoA), and non-metric multidimensional scaling (NMDS) plots, with PCA performed using the stats package (Vienna, Austria), and PCoA and NMDS using photoseq (Seattle, United States). The intergroup differences in β-diversity were assessed using parametric and non-parametric tests, including the Tukey test and Kruskal–Wallis test.

The Linear Discriminant Analysis Effect Size (LEfSe) analysis was conducted with LEfSe (v1.1.2), setting a default linear discriminant analysis (LDA) score filtering value of 3.6. The Metastats analysis (Cambridge, United States) was performed using Mothur to conduct permutation tests at various taxonomic levels (phylum, class, order, family, and genus, species), with *p*-values adjusted using the Benjamin–Hochberg false discovery rate to obtain *q*-values. The analysis of molecular variance (AMOVA) was carried out using the AMOVA function (Geneva, Switzerland) in Mothur (Ann Arbor, United States). Species with significant intergroup differences were analyzed using intergroup *t*-tests and plotted in R.

### 3.3 Measurement of rumen metabolites

#### 3.3.1 Untargeted analysis of rumen metabolites

Untargeted metabolomics of rumen digesta samples (*n* = 6 for each sheep breed) was performed by Wuhan Metware Metabolic Biotechnology Co., Ltd. The results were analyzed using the Metware Cloud platform.

#### 3.3.2 High-performance liquid chromatography (HPLC) conditions

All samples were analyzed using liquid chromatography–mass spectrometry (LC–MS) under both positive and negative ion conditions (Rakusanova and Cajka, 2024). In positive ion mode, the samples were eluted from a Waters ACQUITY Premier HSS T3 Column (Milford, United States) (1.8 μm, 2.1 mm × 100 mm) using a gradient of 0.1% formic acid in water (solvent A) and 0.1% formic acid in acetonitrile (solvent B). The gradient was as follows: 5% solvent B to 20% over 2 min, 20–60% over 3 min, 60–99% over 1 min, held at 99% for 1.5 min, and then returned to 5% solvent B in 0.1 min, holding for 2.4 min. The column was maintained at 40°C, with a flow rate of 0.4 mL/min and an injection volume of 4 μL.

#### 3.3.3 MS conditions (AB)

The data acquisition was performed in information-dependent acquisition (IDA) mode using Analyst TF 1.7.1 software (Sciex, Framingham, USA). The source parameters were set as follows: Ion source gas 1 (GAS1), 50 psi; ion source gas 2 (GAS2), 50 psi; curtain gas (CUR), 25 psi; temperature (TEM), 550°C; declustering potential (DP), 60 V (positive mode) or − 60 V (negative mode); and ion spray voltage floating (ISVF), 5,000 V (positive mode) or − 4,000 V (negative mode).

For the time-of-flight Mass Spectrometry Conditions (Acquisition Bioinformatics) scan, the following parameters were used: mass range, 50–1,000 Da, accumulation time of 200 ms, and dynamic background subtraction enabled. For the product ion scan, the following parameters were used: mass range of 25–1,000 Da, accumulation time of 40 ms, and collision energy set to 30 V (positive mode) or − 30 V (negative mode). The collision energy spread was 15 V, with unit resolution and a charge state of 1. An intensity threshold of 100 cps was used, and isotopes within 4 Da were excluded. A mass tolerance of 50 ppm was enabled, and a maximum of 18 candidate ions were monitored per cycle.

#### 3.3.4 Detection of bile acids

The bile acid metabolomics of rumen digesta samples (*n* = 6 for each sheep breed) was performed by Wuhan Metware Metabolic Biotechnology Co., Ltd. The results were analyzed using the Metware Cloud platform. The bile acid levels were determined by MetWare^4^ using the AB Sciex QTRAP 6500+ LC–MS/MS platform (SCIEX, Redwood, CA, United States).

## 4 Statistical analyses

All the statistical analyses were conducted using GraphPad Prism, v7.0 (GraphPad, La Jolla, CA, USA) or the R package (Wang et al., 2021). Six biologically independent experiments were performed. All the data are presented as the mean ± standard deviation (SD). The data of the basic information of serum parameters, immunoglobulin levels, rumen microbiome *α*-diversity indices of the three groups were analyzed with one-way ANOVA and Student’s *t*-test using SPSS v22.0 (IBM, Chicago, IL, USA) software and asterisks denote statistical significance (*p < 0.05; **p < 0.01; ***p < 0.001).

## 5 Results

### 5.1 Evaluation of health and disease resistance in Hu, DH, and EF sheep using serum immune markers

**Figure 1:**
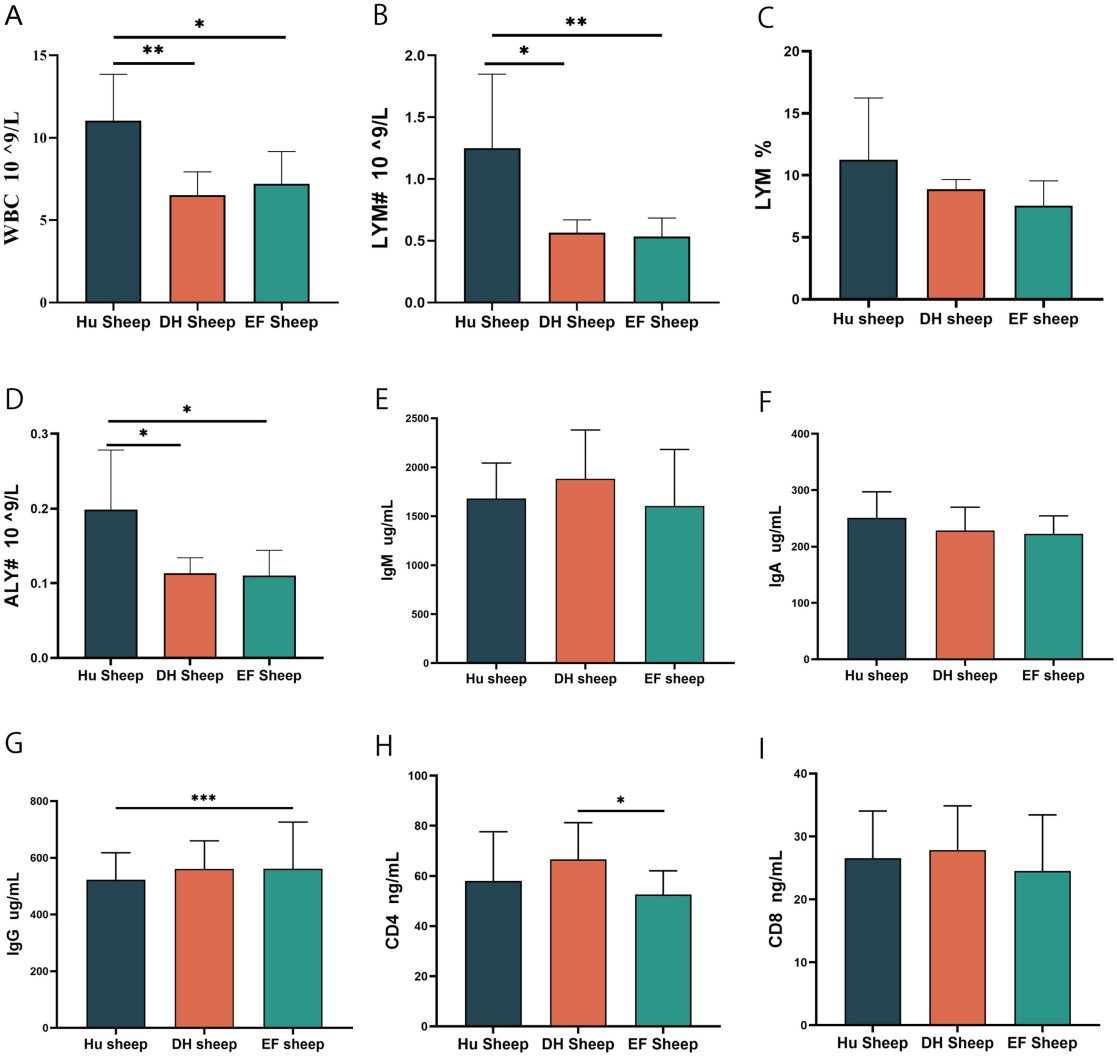
Serum biochemical and immunological parameters of Hu, DH, and EF sheep. **(A)** White blood cell (WBC) count (*n* = 6); **(B)** Iymphocyte count (#LYM, *n* = 6); **(C)** Percentage of lymphocytes (LYM%, *n* = 6). **(D)** Abnormal lymph count (#ALY, *n* = 6); **(E)** Immunoglobulin M (IgM, *n* = 6); **(F)** Immunoglobulin A (IgA, *n* = 6); **(G)** Immunoglobulin G (IgG, *n* = 6); **(H)** CD4 level (CD4, *n* = 6); **(I)** CD 8 level (CD8, *n* = 6). *p < 0.05; **p < 0.01; ***p < 0.001.

To assess the health status and disease resistance of the three sheep breeds, we analyzed serum stress-related immunoglobulins and inflammatory indices. Hu sheep exhibited significantly higher white blood cell (WBC) counts, lymphocyte counts (LYM#), and abnormal lymphocyte counts (ALY#) compared to EF and DH sheep (*p* < 0.05) (Figures 1A,B,D). The percentage of lymphocytes (LYM%) in Hu sheep was also higher, although they were not significantly different (Figure 1C), indicating higher disease resistance.

No significant differences were observed in immunoglobulin M (IgM) and IgA levels (Figures 1E,F). However, the IgG levels in EF sheep were higher than those in Hu sheep (*p* < 0.05), but were not significantly different from DH sheep (Figure 1G). Serum CD4 levels were higher in DH sheep than in EF sheep (*p* < 0.05) (Figure 1H), while CD8+ T cell counts showed no significant differences (Figure 1I). These findings indicate that crossbreeding may enhance disease resistance, providing valuable insights for future breeding strategies.

### 5.2 Characterization of rumen microbiome composition and functional adaptations in Hu, DH, and EF sheep using 16S rRNA and metagenomic sequencing

A total of 2,115,281 16S rRNA gene tags were generated from 18 rumen digesta samples (*n* = 6 for each sheep breed), resulting in 14,430 amplicon sequence variants (ASVs). Rank abundance, rarefaction, and species accumulation curves indicated sufficient sequencing depth (Supplementary Figure S1; Wang et al., 2020) and illustrated the distribution of the top 19 operational taxonomic units (OTUs) by phylum. The rumen microbiome composition in all breeds was dominated by *Bacteroidetes*, *Firmicutes*, and *Actinobacteria*, exhibiting specific variations. In Hu sheep, the predominant phyla were *Bacteroidetes* (47%), *Firmicutes* (45%), *Actinobacteria* (3%), and Spirochaetota (2%) (Supplementary Figure S2A). In DH sheep, *Bacteroidetes* (55%) and *Firmicutes* (41%) predominated, while EF sheep exhibited the highest abundance of *Firmicutes* (55%) and *Proteobacteria* (3%) (Supplementary Figures S2B,C).

**Figure 2:**
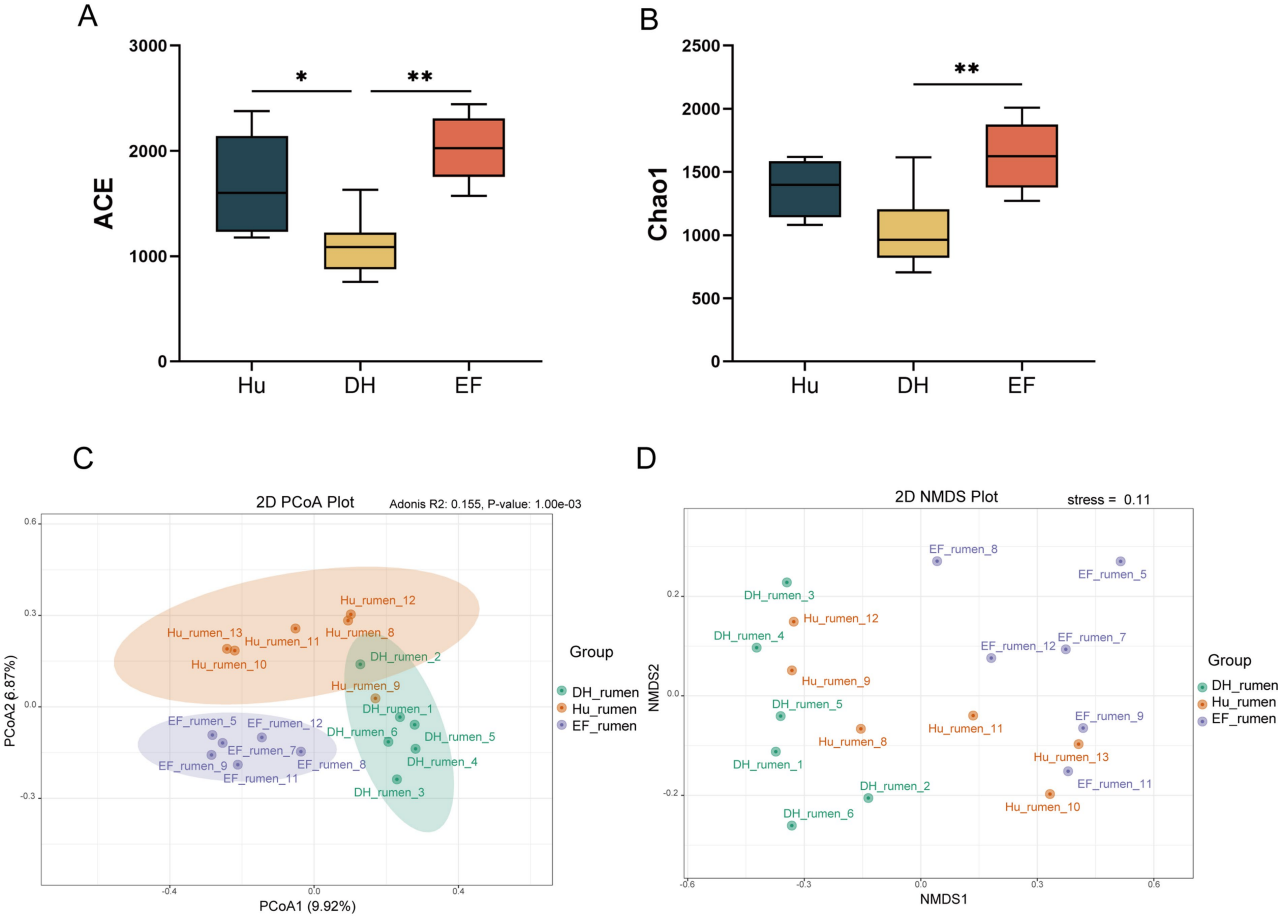
Diversity analysis of rumen microbiome in Hu, DH, and EF Sheep. **(A)** ACE index; **(B)** Chao1 index; **(C)** PCoA of the ruminal microbiome; **(D)** NMDS analysis of the ruminal microbiome.

Significant differences in *α*-diversity indices were observed. Hu and EF sheep exhibited higher ACE indices than DH sheep (*p* < 0.05) (Figure 2A), and the Chao1 index of EF sheep was also higher (*p* < 0.05) (Figure 2B). No significant differences were found in Simpson and Shannon indices (Supplementary Figures S1E,F). *β*-Diversity analysis using PCoA and NMDS showed distinct separation between the microbiomes of Hu, DH, and EF sheep (Figures 2C,D). The largest dissimilarity was between Hu and DH sheep, indicating more pronounced differences in their rumen microbiomes.

Among the 14,374 ASVs identified, 3,811 were specific to DH sheep, 3,813 were specific to Hu sheep, and 5,733 were specific to EF sheep (Supplementary Figure S3A). Notably, EF sheep exhibited a higher abundance of *Firmicutes*, *Proteobacteria*, and *Fusobacteriota*, while Hu sheep showed a higher abundance of *Spirochaetota* and *Fibrobacterota*. DH sheep exhibited elevated levels of *Bacteroidota*, *Euryarchaeota*, and Actinobacteria (Supplementary Figure S3B). At the genus level, significant differences were observed. EF sheep exhibited higher abundances of *Lachnospiraceae*, *Veillonellaceae*, *Asteroleplasma*, *Dialister*, and *Blautia* (Supplementary Figures S3C–G), whereas Hu sheep exhibited higher levels of *Acidaminococcaceae*, *Selenomonadaceae*, and *Acetitomaculum*. DH sheep exhibited higher levels of *Streptococcaceae*, *Rikenellaceae*, and *Streptococcus* (*p* < 0.05) (Supplementary Figures S3C–G).

Methane-producing bacteria, such as *Methanobacteriaceae*, *Methanobrevibacter*, and *Methanobacteriales*, were most abundant in DH sheep, followed by Hu sheep, and least abundant in EF sheep (*p* < 0.05) (Supplementary Figures S3C–G). This suggests that DH sheep possess the highest potential for methane production, which is significant for carbon-neutral strategies and hybrid breeding.

**Figure 3:**
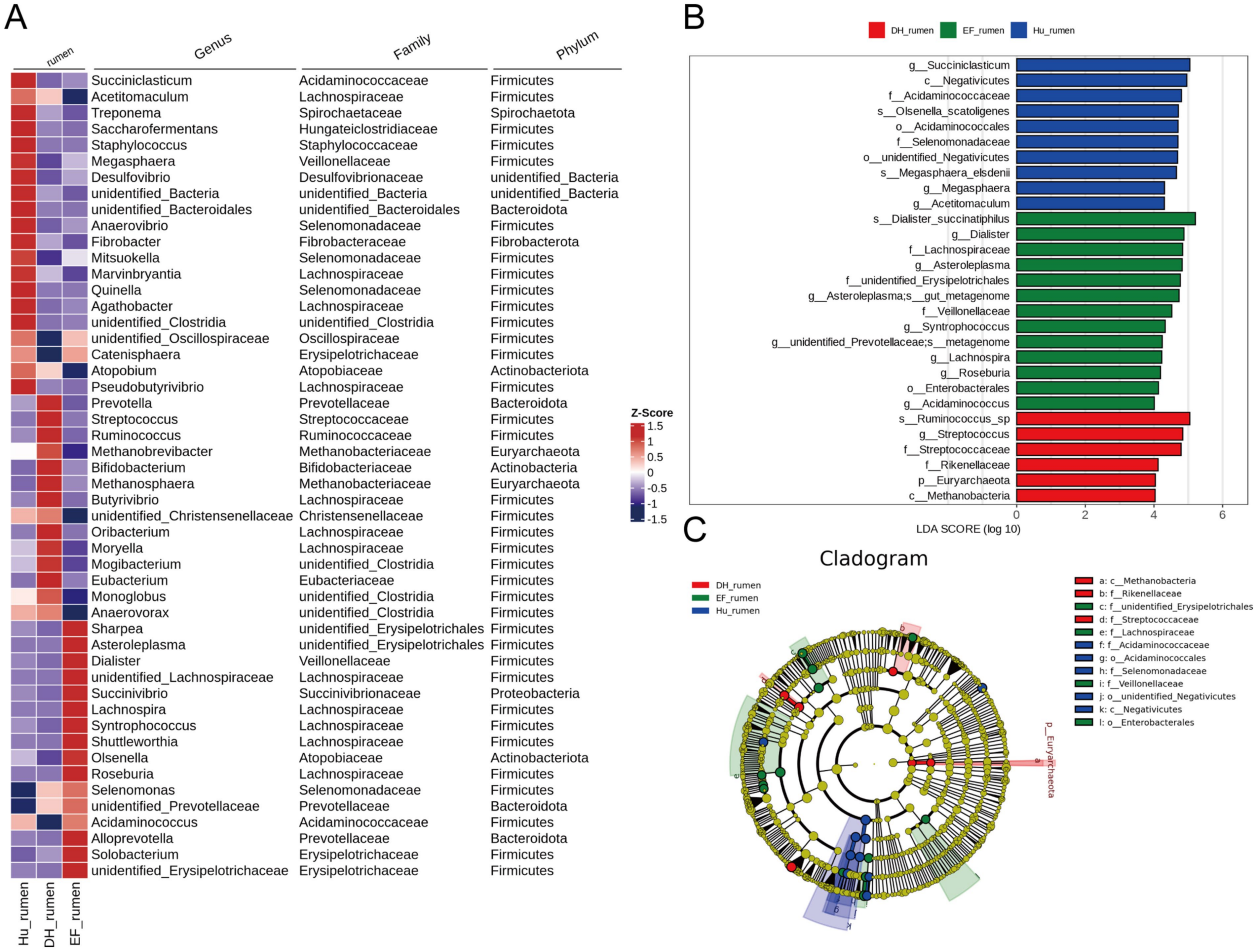
Differential taxonomic composition of the rumen microbiome in Hu, DH, and EF sheep revealed by 16S rRNA sequencing. **(A)**. Genus levels. Heatmap showing the abundance of the top 50 rumen microbes at the genus level; **(B)** and **(C)** Linear discriminant analysis (LDA) effect size (LEfSe) of the rumen microbiome of Hu, DH, and EF sheep. Cladogram plot of differentially abundant species in the three groups. Significant differences were evaluated by LEfSe analysis, with LDA scores above 4 and *p*-values below 0.05. Red indicates enriched taxa in the DH sheep group; green indicates enriched taxa in the EF sheep; blue indicates enriched taxa in the Hu sheep.

LEfSe analysis revealed distinct rumen microorganisms in each breed. In Hu sheep, species such as *Succiniclasticum*, *Acidaminococcaceae*, *Olsenella scatoligenes*, *Megasphaera*, and *Acetitomaculum* were more abundant than in other groups (*p* < 0.05, LDA > 4) (Figures 3A,B). EF sheep exhibited higher levels of *Dialister succinatiphilus*, *Dialister*, *Lachnospiraceae*, and *Asteroleplasma* (*p* < 0.05, LDA > 4) (Figures 3A,B). DH sheep showed higher abundances of *Ruminococcus* sp., *Streptococcus*, *Streptococcaceae*, *Rikenellaceae*, and *Methanobacteria* (*p* < 0.05, LDA > 4) (Figures 3A,B). The branch evolution map showed that key microbial groups in DH sheep included *Rikenellaceae*, *Methanobacteria*, and *Streptococcaceae*, while EF sheep contained *Erysipelotrichales*, *Lachnospiraceae*, and *Veillonellaceae*, and Hu sheep featured *Acidaminococcaceae*, *Selenomonadaceae*, and *Negativicutes* (Figure 3C).

**Figure 4:**
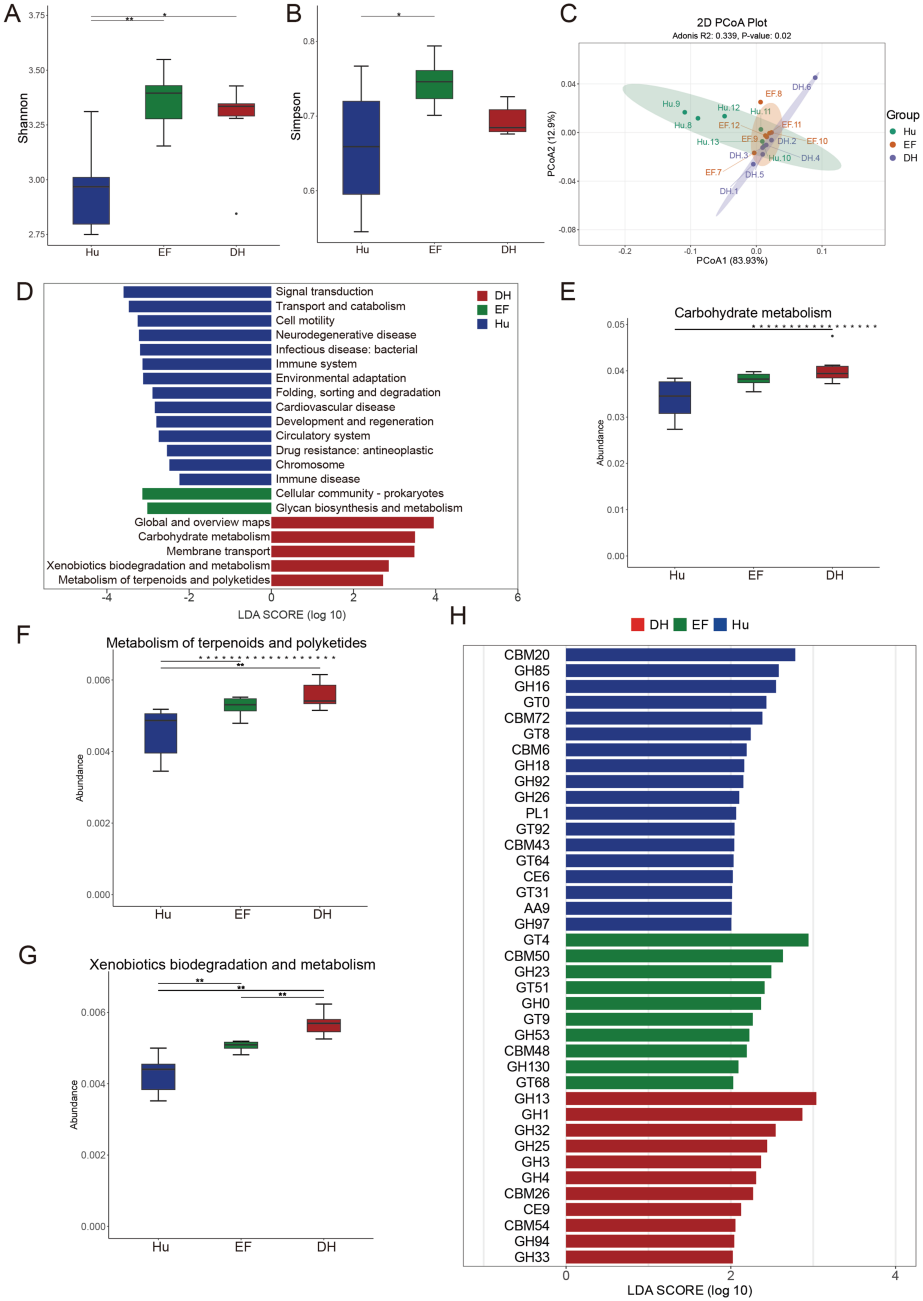
Comparative analysis of rumen microbiome diversity and function among Hu, DH, and EF sheep. **(A)** Shannon index of *α*-diversity; **(B)** Simpson index of α-diversity; **(C)** PCoA analysis of KEGG level-2 functional profiles based on metagenomic sequencing; **(D)** LEfSe analysis of KEGG level-2 pathways in the rumen microbiome; **(E)** Carbohydrate metabolism pathway; **(F)** Metabolism of terpenoids and polyketides; **(G)** Xenobiotic biodegradation and metabolism, which was assessed using the Kruskal–Wallis test; **(H)** LEfSe analysis of Carbohydrate-Active EnZymes (CAZymes) in the three groups. Significant differences were identified using LEfSe analysis, with an LDA score above 2 and a *p*-value below 0.05. Red indicates taxa enriched in DH sheep; green indicates taxa enriched in EF sheep; and blue indicates taxa enriched in Hu sheep.

16S rRNA gene sequencing is a valuable method for revealing the composition of microbial communities; however, its resolution may not be sufficient for analyzing specific functional genes. Therefore, we employed metagenomic sequencing technology to further investigate functional genes associated with growth, immunity, and metabolism. A total of 152.41 Gb of clean data were generated from 18 rumen digesta samples (*n* = 6 for each sheep breed) (Supplementary Table S7). Significant differences in *α*-diversity indices were observed. The Shannon indices of DH and EF sheep were significantly higher than those of Hu sheep (*p* < 0.05) (Figure 4A), while the Simpson index of EF sheep was also significantly higher than that of Hu sheep (*p* < 0.05) (Figure 4B). However, no significant differences were observed in the ACE and Chao1 indices (Supplementary Figures S4A,B).

*β*-diversity analysis using PCoA and NMDS demonstrated a clear separation of microbiomes among the Hu, DH, and EF sheep (Supplementary Figures S4C,D). To further identify differential microbial taxa, we applied a more stringent criterion (LDA > 4). In the rumen of Hu sheep, *Prevotella*, *Hallella*, and *Hallella mizrahii* were significantly enriched. In contrast, the EF sheep rumen exhibited a significant enrichment of *Roseburia*, *Intestinibaculum*, and *Intestinibaculum porci*. In DH sheep, *Streptococcus*, *Sarcina* sp. *DSM-11001*, *Sarcina*, *Ruminococcus*, *Streptococcus equinus*, and particularly *Methanobrevibacter*, *Methanobacteriaceae*, *Methanobacteria*, and *Methanobacteriales* were significantly enriched (Supplementary Figure S4E). Furthermore, the microbial composition at the phylum, family, and genus levels in the Hu, EF, and DH sheep rumen was consistent with previous findings (Supplementary Figures S4F–I), further validating our results.

We, subsequently, performed the Kyoto Encyclopedia of Genes and Genomes (KEGG) functional analysis on the metagenomic data, revealing significant differences among the three sheep breeds at KEGG level 2 based on PCoA analysis (Figure 4C). LefSE analysis at KEGG level 2 indicated that the predominant microbial functions in DH sheep rumen were enriched in carbohydrate metabolism, membrane transport, xenobiotic biodegradation and metabolism, and the metabolism of terpenoids and polyketides (Figure 4D). These enriched pathways suggest that DH sheep possess a metabolically versatile rumen microbiota, potentially contributing to enhanced energy utilization, efficient xenobiotic degradation, and secondary metabolite processing. Further Kruskal–Wallis test analysis of these pathways confirmed that DH sheep exhibited significantly higher activity than EF and Hu sheep (Figures 4E–G), indicating that DH sheep may have developed an adaptive advantage in rumen metabolic function through crossbreeding.

Finally, Carbohydrate-Active EnZymes (CAZyme) analysis demonstrated that Hu sheep had the highest abundance of CAZyme, followed by DH sheep, while EF sheep exhibited the lowest abundance (Figure 4H). This finding indicates that the rumen microbiota of Hu sheep possesses a strong carbohydrate degradation capability, which has been partially transmitted to DH hybrid sheep, thereby compensating for the limited carbohydrate enzyme activity observed in EF sheep. The combined results highlight the distinct metabolic adaptations among the three breeds, with DH sheep exhibiting a unique balance of microbial functions that may provide enhanced rumen efficiency and environmental adaptability.

### 5.3 Characterization of metabolic profiles in rumen samples from Hu, DH, and EF sheep reveals distinct bile acid and fatty acid signatures using LC–MS-based metabolomics

**Figure 5:**
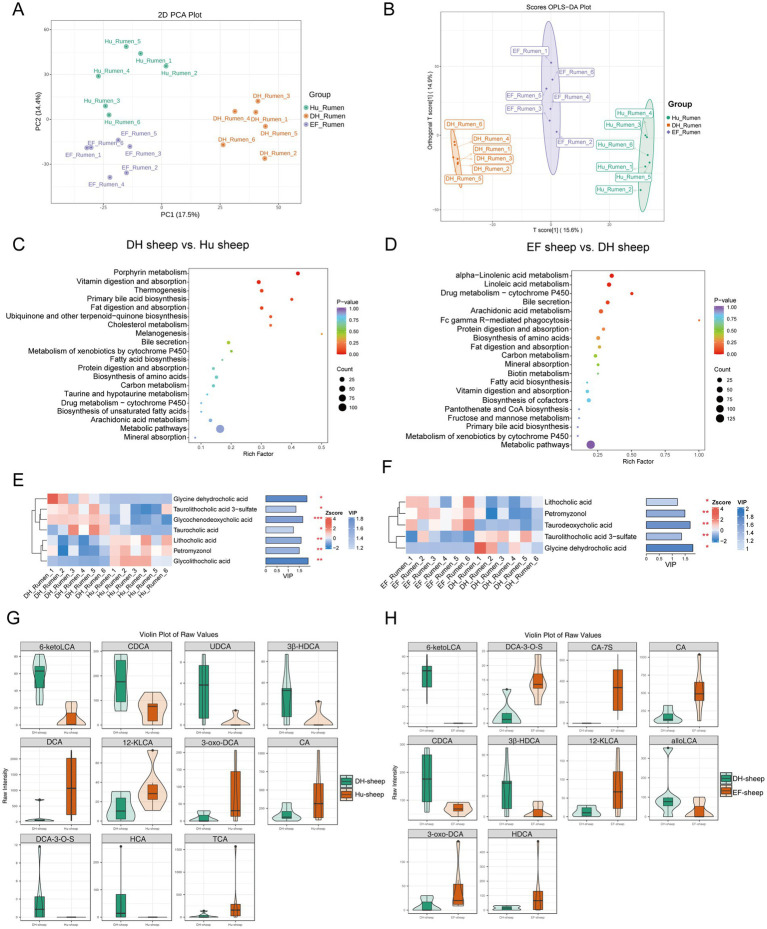
Characterization of significantly differentially abundant rumen metabolites in Hu, DH, and EF Sheep. **(A)** Principal component analysis (PCA) of the ruminal metabolites of Hu, DH, and EF sheep; **(B)** Non-metric multidimensional scaling (NMDS) of the ruminal metabolites of Hu, DH, and EF sheep. **(C, D)** KEGG signaling pathways enriched in differentially abundant ruminal metabolites between DH and Hu sheep and between DH and EF sheep. **(E, F)** Variable importance in the projection (VIP) score analysis for differential secondary bile acid metabolites. Bile acids with VIP scores above 1 were selected and ranked based on VIP scores. The length of the bar indicates the value of the contribution of this metabolite to the difference between the two groups. The color of the bar indicates the significance of the difference between the two groups of samples. *p < 0.05; **p < 0.01; ***p < 0.001. **(G, H)** Bile acid-targeted metabolic violin plots. All selected plots had bile acid *p*-values below 0.05.

The total ion chromatograms (TICs) of the QC samples in both positive and negative ion modes showed overlapping peaks and consistent retention times (Supplementary Figure S5), thereby confirming the data reliability. To assess overall differences and variability among rumen samples, PCA and orthogonal partial least squares discriminant analysis (OPLS-DA) were performed (Figure 5A). Both analyses revealed significant differences among Hu, DH, and EF sheep (Figure 5B). The models had R^2^Y-values above 0.9 and Q^2^-values above 0.7, demonstrating strong predictive ability (Supplementary Figure S6).

Metabolites were classified into 26 compound groups, with the top 5 representing 64% of the total metabolites (Supplementary Figure S7A). Volcano plots identified differentially abundant metabolites between EF and DH sheep, as well as between DH and Hu sheep (Supplementary Figures S7B,C). Heatmaps highlighted significant differences in 1097 metabolites between DH and Hu sheep (Supplementary Figure S6D) and 1,190 metabolites between DH and EF sheep (Supplementary Figure S7E). The KEGG enrichment analysis revealed pathways related to bile secretion, fat digestion and absorption, and primary bile acid biosynthesis (Figures 5C,D).

Differentially abundant bile acids were also identified. Glycine dehydrocholic acid, taurolithocholic acid 30-sulfate, glycochenodeoxycholic acid, and taurocholic acid were more abundant in DH sheep compared to Hu sheep (*p* < 0.05), while lithocholic acid, petromyzonol, and glycolithocholic acid were more abundant in Hu sheep (Figure 5E). DH sheep exhibited significantly higher levels of taurolithocholic acid 3-sulfate and glycine dehydrocholic acid compared to EF sheep, while lithocholic acid, petromyzonol, and taurodeoxycholic acid were more abundant in EF sheep (*p* < 0.05) (Figure 5F).

Targeted metabolomics confirmed these findings, showing significantly higher levels of 6-keto lithocholic acid (6-ketoLCA), cholic acid and chenodeoxycholic acid (CDCA), 6-keto lithocholic acid (6-ketoLCA), cholic acid and chenodeoxycholic acid (CDCA), ursodeoxycholic acid (UDCA), 3β-hyodeoxycholic acid (3β-HDCA), divisive cluster analysis (DCA)-3-O-S, and hierarchical cluster analysis (HCA) in DH sheep compared to Hu sheep (*p* < 0.05), whereas DCA, 12-keto lithocholic acid (12-KLCA), 3-oxo-DCA, cholic acid (CA), and taurine-conjugated bile acid (TCA) were higher in Hu sheep (Figure 5G). DH sheep also had higher levels of 6-ketoLCA, CDCA, 3β-HDCA, and AlaloLCA than EF sheep (*p* < 0.05), while EF sheep exhibited higher levels of DCA-3-O-S, CA-7S, CA, 12-KLCA, 3-oxo-DCA, and HDCA (Figure 5H). Notably, 6-ketoLCA, CDCA, and 3β-HDCA were significantly higher in DH sheep compared to both Hu and EF sheep (Supplementary Figure S8).

To explore the impact of bile acids on fatty acid profiles, we found that trans, trans-muconic acid, cis-epoxyoctadecanoic acid (cis-EODA), cis, cis-muconic acid, and stearic acid were more abundant in Hu sheep than in DH sheep (*p* < 0.05) (Supplementary Figure S9A). Conversely, DH sheep exhibited significantly higher levels of 17-octadecynoic acid, undecanoic acid, 6-hydroxypentadecanedioic acid, traumatic acid, 2-benzylsuccinic acid, sebacic acid, and oleoyltaurine compared to Hu sheep (*p* < 0.05). DH sheep also had higher levels of FFAs (20: 0), 10-nitrooleate, oleoyltaurine, sebacic acid, stearidonic acid, FFAs (16:1), undecanoic acid, 17-octadecynoic acid, and 2-benzylsuccinic acid compared to EF sheep, while 9,10-epoxystearic acid, FFAs (18:2), cis-EODA, uric acid, and trans, trans-medic acid were lower (*p* < 0.05) (Supplementary Figure S9B).

### 5.4 Characterization of serum metabolic profiles in Hu, DH, and EF sheep discovers distinct bile acid signatures and pathway enrichments via LC–MS-based non-targeted metabolomics

**Figure 6:**
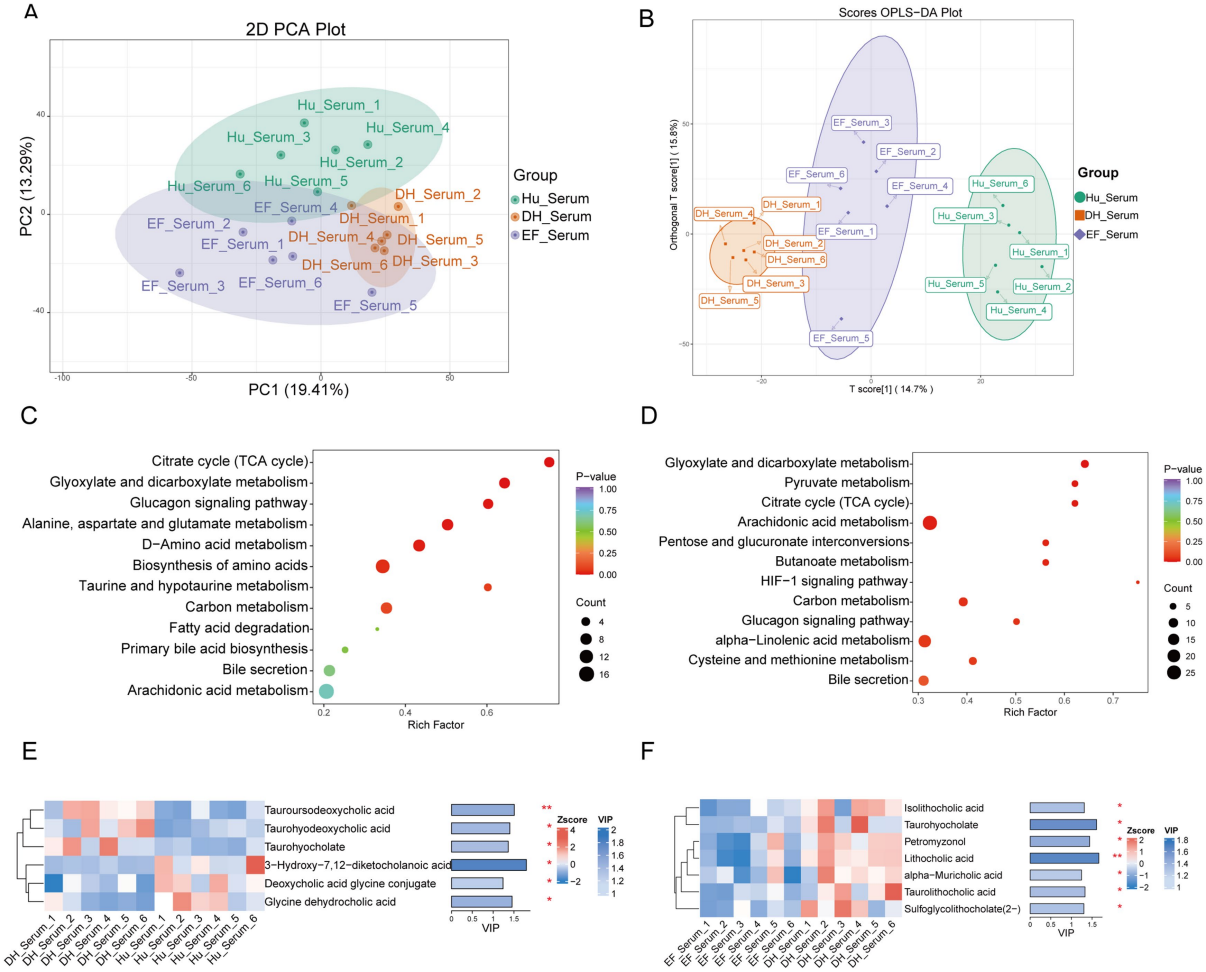
Significantly different serum metabolites of Hu, DH, and EF sheep. **(A)** Principal component analysis (PCA) of the serum metabolites of Hu, DH, and EF sheep. **(B)** Non-metric multidimensional scaling (NMDS) of the serum metabolites of Hu, DH, and EF sheep. **(C, D)** KEGG signaling pathways enriched in the differentially abundant serum metabolites of DH vs. Hu sheep and DH vs. EF sheep. **(E, F)** Variable importance in the projection (VIP) score analysis for differentially abundant secondary bile acid metabolites. Bile acids with VIP scores above 1 were selected and ranked based on VIP scores. *p < 0.05; **p < 0.01; ***p < 0.001.

To explore the colonic metabolic composition, we employed a LC–MS-based untargeted metabolomics approach (Supplementary Figure S10). Unsupervised principal component analysis (PCA) revealed clear separation of serum metabolites from Hu sheep, DH sheep, and EF sheep, indicating distinct metabolic profiles (Figure 6A). Supervised orthogonal partial least squares discriminant analysis (OPLS-DA) further highlighted significant differences among the three groups (*p* < 0.05) (Figure 6B). The model parameters R^2^Y were higher than 0.99, and Q^2^-values exceeded 0.7, confirming the models’ excellent predictive ability (Supplementary Figures S11A–C). These results demonstrate substantial metabolic differences in the serum of Hu, DH, and EF sheep.

Metabolites were categorized into 26 compound groups, with the top five groups accounting for 63.4% of the total metabolites (Supplementary Figure S12A). Volcano plots illustrated the differentially abundant metabolites between EF and DH sheep, as well as between DH and Hu sheep (Supplementary Figures S12B,C). Heatmaps based on 611 and 628 differentially abundant metabolites showed distinct differences between DH and Hu sheep, as well as between DH and EF sheep, respectively (Supplementary Figures 12D,E). The KEGG enrichment analysis identified six enriched pathways: the citrate cycle (TCA cycle), glyoxylate and dicarboxylate metabolism, glucagon signaling, biosynthesis of amino acids, alanine-aspartate–glutamate metabolism, and D-amino acid metabolism (Figures 6C,D).

Notably, bile acid levels in the serum showed significant differences. Taurohyodeoxycholic acid, taurohyodeoxycholic acid, and taurohyocholate were more abundant in DH sheep serum compared to Hu sheep serum (*p* < 0.05), while 3-hydroxy-7,12-diketocholanoic acid, deoxycholic acid glycine conjugate, and glycine dehydrocholic acid were more abundant in Hu sheep serum (*p* < 0.05) (Figure 6E). Additionally, several bile acids, including isololithocholic acid, taurohyocholate, petromyzonol, lithocholic acid, *α*-muricholic acid, taurolithocholic acid, and sulfoglycolithocholate (2-), were more abundant in DH sheep serum than in EF sheep serum (*p* < 0.05) (Figure 6F).

## 6 Bile acid metabolite–microbiota and immunomicrobiota correlations in rumen and serum of different sheep breeds

### 6.1 Rumen bile acid metabolite–microbiota correlations

**Figure 7:**
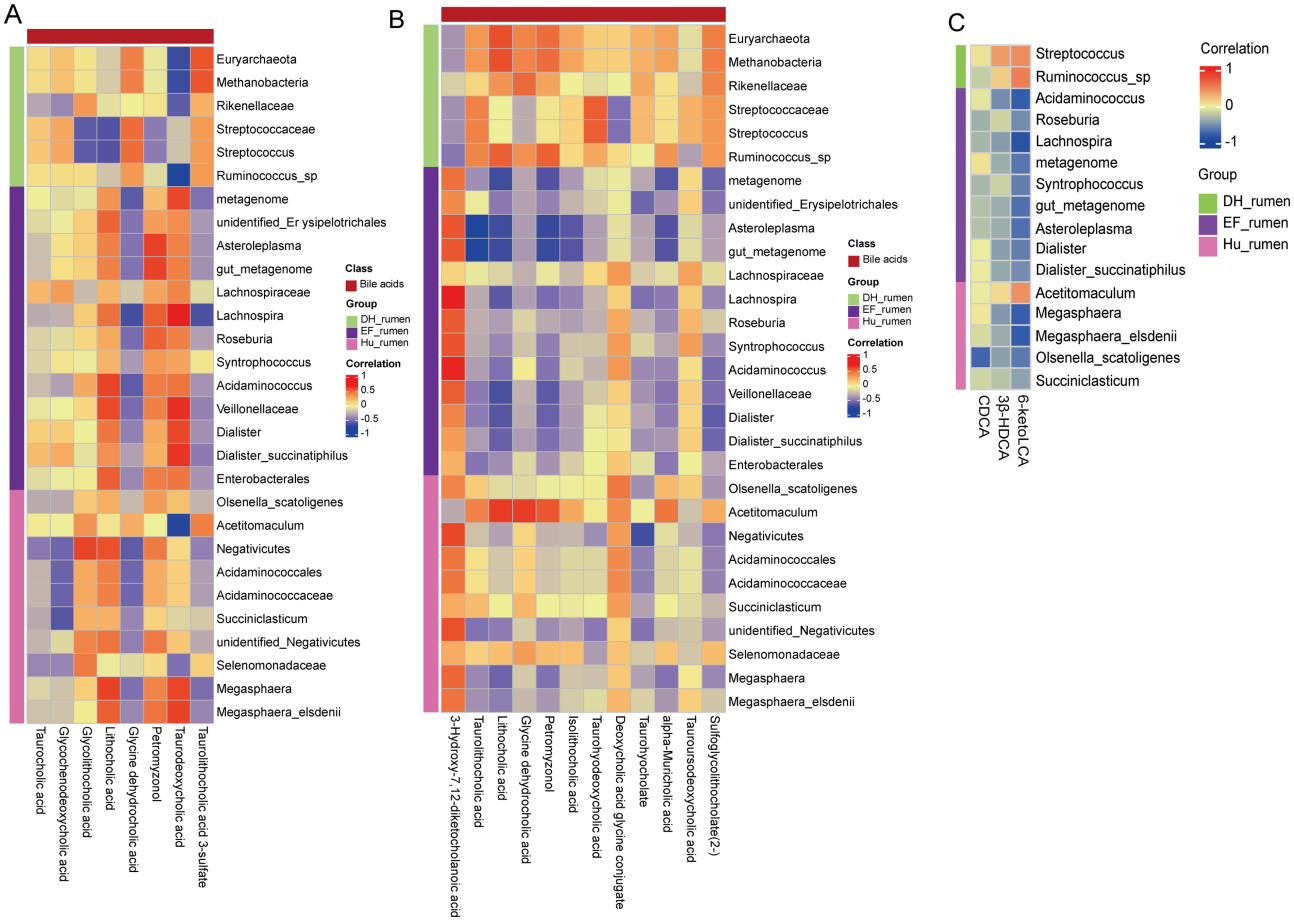
Correlation analysis of differentially abundant bile acid metabolites and the rumen microbiome. Each row represents a ruminal microbe and each column represents bile acid metabolites. Red indicates a positive correlation; blue indicates a negative correlation. **(A)** Spearman correlation analysis between rumen metabolites and rumen microorganisms. **(B)** Spearman correlation analysis between serum metabolites and rumen microorganisms. **(C)** Spearman correlation analysis between targeted rumen metabolites and rumen microorganisms.

In the DH sheep group, *Streptococcus* exhibited a weak positive correlation with taurocholic acid (r = 0.060, *p* = 0.810) and CDCA (r = 0.060, *p* = 0.810), while *Ruminococcus* sp. showed a weak negative correlation with taurocholic acid (r = 0.184, *p* = 0.466) and CDCA (r = 0.184, *p* = 0.466). Both taxa displayed positive correlations with taurodeoxycholic acid (*Streptococcus*: r = 0.399, *p* = 0.101; *Ruminococcus* sp.: r = 0.161, *p* = 0.523) and 3β-HDCA (*Streptococcus*: r = 0.399, *p* = 0.101; *Ruminococcus* sp.: r = 0.161, *p* = 0.523). Notably, *Streptococcus* (r = 0.483, *p* = 0.042) and *Ruminococcus* sp. (r = 0.543, *p* = 0.020) exhibited significant positive correlations with taurolithocholic acid 3-sulfate and 6-ketoLCA. In the EF sheep group, multiple taxa exhibited significant negative correlations with taurolithocholic acid 3-sulfate and 6-ketoLCA, including *Acidaminococcus* (r = −0.775, *p* = 0.00016), *Roseburia* (r = −0.525, *p* = 0.025), *Lachnospira* (r = −0.854, *p* = 6.404 × 10^−6^), metagenomic features (r = −0.632, *p* = 0.005), *Syntrophococcus* (r = −0.590, *p* = 0.010), gut_metagenome (r = −0.672, *p* = 0.002), *Asteroleplasma* (r = −0.671, *p* = 0.002), *Dialister* (r = −0.590, *p* = 0.010), and *D. succinatiphilus* (r = −0.520, *p* = 0.027). In the Hu sheep group, *O. scatoligenes* exhibited a significant negative correlation with taurocholic acid (r = −0.728, *p* = 0.0006) and CDCA (r = −0.728, *p* = 0.0006). *Acidaminococcus* (r = −0.524, *p* = 0.025) and *Megasphaera* (r = −0.473, *p* = 0.048) were significantly negatively correlated with glycocholic acid and 3β-HDCA. Additionally, *Megasphaera* (r = −0.760, *p* = 2.543 × 10^−4^), *Megasphaera elsdenii* (r = −0.786, *p* = 1.091 × 10^−4^), and *O. scatoligenes* (r = −0.616, *p* = 0.006) exhibited significant negative correlations with taurolithocholic acid 3-sulfate and 6-ketoLCA (Figures 7A,C).

### 6.2 Serum bile acid metabolite–microbiota correlations

In the serum, distinct correlation patterns were also observed. For example, in DH sheep, *Acetitomaculum* was significantly positively correlated with glycine dehydrocholic acid (r = 0.651, *p* = 0.003) and lithocholic acid (r = 0.655, *p* = 0.003), and it also showed a positive association with petromyzonol (r = 0.573, *p* = 0.013). Meanwhile, *Acidaminococcus* exhibited a significant positive correlation with 3-hydroxy-7,12-diketocholanoic acid (r = 0.684, *p* = 0.002) and significant negative correlations with lithocholic acid (r = −0.536, *p* = 0.022) and sulfoglycolithocholate (2–) (r = −0.556, *p* = 0.017). Similar trends were noted for other taxa (e.g., *Asteroleplasma*, *Dialister*, and the gut metagenome), indicating a complex interplay between the rumen microbial community and serum bile acid profiles (Figure 7B).

### 6.3 Immune indicator–microbiota correlations

In DH sheep, *Ruminococcus* sp. (r = 0.479, *p* = 0.044) and *Rikenellaceae* (r = 0.511, *p* = 0.030) exhibited significant positive correlations with IgA, indicating a stronger mucosal immune response. Additionally, *Methanobacteria* and *Euryarchaeota* showed moderate positive associations with CD4+ T cells, indicating potential support for adaptive immunity (Supplementary Figure S13A). In Hu sheep, *Lachnospiraceae* was negatively associated with CD4+ T cells (r = −0.439, *p* = 0.068), and unidentified *Erysipelotrichales* exhibited a trend toward a negative correlation with IgA (r = −0.371, *p* = 0.130) (Supplementary Figure S13B). These findings indicate a distinct immune indicator–microbiota relationship in Hu sheep compared to DH and EF breeds. In EF sheep, *Syntrophococcus* displayed a strong negative correlation with IgM (r = −0.699, *p* = 0.001), suggesting a possible immunoregulatory role. *D. succinatiphilus* was negatively associated with IgA (r = −0.479, *p* = 0.044), implying a potential influence on antibody-mediated immunity (Supplementary Figure S13C).

Overall, DH sheep demonstrated stronger positive immune indicator–microbiota correlations, particularly with IgA, suggesting enhanced mucosal immunity. EF and Hu sheep showed more negative associations, indicating potential differences in immune regulation and microbial influences across breeds.

## 7 Discussion

This study provides a comprehensive comparison of immune responses, microbiome compositions, and metabolomic profiles via three sheep breeds—Hu, DH, and EF. The findings underscore significant breed-specific differences, which not only highlight the intrinsic genetic variation among these breeds but also emphasize the role of microbial and metabolic factors in influencing immune responses and overall health. Notably, crossbreeding effects between Hu and DH sheep are evident, particularly in immune function, rumen microbiome diversity, and metabolite profiles.

A major observation from this study is the significant variation in immune responses between the breeds. The lymphocyte count in Hu sheep was notably higher compared to DH and EF sheep, suggesting that Hu sheep may have a stronger basal immune capacity. T lymphocytes, crucial for cell-mediated immunity, help eliminate infected cells by directly recognizing them (Matsushita et al., 2015; Swain et al., 2012). Our results are consistent with this, where Hu sheep exhibited more pronounced immune activity. These differences in immune markers align with findings from previous studies that indicate breed-specific variations in immune function.

One of the key immune factors was the CD4+/CD8+ T lymphocyte ratio (Zhang and Bevan, 2011), which serves as an indicator of immune health (Rey-Jurado et al., 2020). Our results showed that DH sheep, despite having a lower lymphocyte count than Hu sheep, exhibited superior immune responses (Ojha et al., 2020). This is further supported by a higher CD4/CD8+ T cell ratio in DH sheep compared to EF sheep, suggesting that DH sheep might be more capable of mounting an effective immune response against pathogens. These findings align with previous research indicating that the immune capacity of DH sheep is better than that of EF sheep in terms of immune factors (Ruterbusch et al., 2020; Chai et al., 2020; Yang et al., 2022).

The rumen microbiome plays a key role in animal health, affecting digestion, metabolism, and immune function. Our analysis revealed significant variations in ruminal microbiome composition among the three sheep breeds. Despite these differences, all breeds shared core microbiome features, such as *Firmicutes*, *Bacteroidetes*, and *Actinobacteria*, consistent with previous reports (Liu et al., 2016; Jin et al., 2018; Huws et al., 2016). However, distinct microbial signatures were found in DH sheep, including higher relative abundances of *Prevotella*, *Succiniclasticum* (Koike et al., 2021), and *Acetitomaculum* (Wang et al., 2016; Wu et al., 2011). These genera are known for their role in cellulose degradation, with *Prevotella* being associated with rumen maturation and growth in ruminants (Jiao et al., 2015; Rey et al., 2014; Koringa et al., 2019). Interestingly, *Prevotella* was more abundant in DH sheep, suggesting that DH sheep may have a more mature and efficient rumen microbiome (Hart et al., 2018; Snelling and Wallace, 2017; Moraïs and Mizrahi, 2019), which could enhance growth and boost disease resistance (Jiao et al., 2015; Kitanaka et al., 2019). Our findings indicate that the enrichment of *Prevotella* could be a key factor in the superior growth rates and disease resistance observed in DH sheep, underscoring the potential benefits of crossbreeding Hu and DH sheep for enhanced rumen development. Additionally, we observed higher levels of *Macrococcus* in Hu sheep compared to the other breeds. This bacterium has been shown to alleviate ruminal acidosis, and its higher abundance in Hu sheep could explain their potential resilience to this condition (Chai et al., 2021; Monteiro et al., 2022). These microbial differences between breeds suggest that certain microbial species may positively impact health and resilience and enhance digestive disorders, and these benefits could be influenced by genetic factors, including those from crossbreeding (Gu et al., 2023).

One of the most striking findings from our study was the variation in rumen metabolites across the three sheep breeds, particularly in bile acid profiles. Bile acids, such as 6-ketoLCA, CDCA, and 3β-HDCA, were significantly more abundant in DH sheep than in Hu and EF sheep. These bile acids have crucial roles in maintaining gut and rumen health (Song et al., 2019), with CDCA promoting intestinal health and regulating the immune response in pigs (Xu et al., 2022) and goats (Jin et al., 2024). The higher levels of CDCA in DH sheep likely contribute to maintaining the integrity of the rumen epithelium and enhancing immune responses, thus improving disease resistance. Furthermore, the increased levels of 3β-HDCA in DH sheep support the idea that bile acids can reduce systemic inflammation and promote organ health (Geng et al., 2022; She et al., 2024). HDCA has been shown to alleviate conditions like non-alcoholic fatty liver disease (NAFLD) and sepsis by reducing inflammatory responses (Kuang et al., 2023; Li et al., 2023). The significant elevation of 3β-HDCA in DH sheep might explain the breed’s superior disease resistance at the metabolic level. These findings highlight the potential of bile acids as biomarkers for breed-specific health benefits and indicate that DH sheep, with their enhanced bile acid profiles, may have an intrinsic advantage in terms of immune function and disease resistance. Finally, isolithocholic acid and taurolithocholic acid were significantly elevated in DH sheep, suggesting that these bile acids confer metabolic advantages. Isolithocholic acid has been shown to reduce colitis symptoms in mice (Kubota et al., 2023), while Taurolithocholic acid plays a key role in regulating bile acid circulation and cholesterol metabolism, potentially influencing overall metabolic health (Sun et al., 2023; Lowjaga et al., 2021). These elevated bile acid levels in DH sheep may contribute not only to digestive health but also to enhanced systemic immune function, supporting the breed’s higher disease resistance.

The comparison of DH sheep to Hu and EF sheep reveals the impact of crossbreeding on both immune responses and metabolic profiles. DH sheep not only showed superior immune function but also exhibited distinct differences in their rumen microbiome and bile acid composition. These crossbreeding effects may help to explain the improved health and disease resistance observed in DH sheep compared to the other breeds. The combined genetic and microbiome factors likely work synergistically to enhance the overall resilience of DH sheep, making them a promising candidate for breeding programs aimed at improving disease resistance and growth efficiency in sheep populations. DH sheep exhibited significantly enhanced immune function, characterized by higher lymphocyte counts and elevated IgA levels, indicating a robust immune system. This advantage suggests that using DH sheep as a genetic foundation in crossbreeding can improve disease resistance in hybrid offspring. Collectively, these traits make DH sheep an ideal choice for breeding programs aimed at developing high-performance, disease-resistant livestock, providing a strong foundation for future genetic improvement strategies.

Given the elevated levels of Prevotella in DH sheep, optimizing dietary fiber content or supplementing with specific probiotics could further enhance nutrient utilization and growth performance. The differences in metabolic pathways observed in hybrid sheep highlight the need for tailored nutritional strategies. The significant associations identified between rumen microbiota, metabolites, and immune parameters (Figure 7) suggest that microbiota-targeted dietary interventions could further improve metabolic efficiency. Previous studies have demonstrated that substituting 33% barley starch for corn starch in the diet of Hu sheep does not alter rumen fermentation patterns, but it increases the richness and diversity of the rumen microbiota (Wang et al., 2024). This finding can serve as a reference for future feeding strategies. Additionally, the inclusion of compound probiotics has been shown to improve the production performance of Hu sheep, reduce inflammation, and enhance rumen and intestinal health (Wang et al., 2024). This provides valuable insights for the future integration of specific beneficial core microbial communities from DH hybrid sheep into the parental stock or other sheep breeds to boost their production performance. Additionally, the higher concentrations of bile acids suggest that formulating high-energy feeds may improve metabolic efficiency, maximizing the benefits of their superior digestive capacity. The strong immune response observed in DH sheep indicates that crossbreeding can reduce reliance on antibiotics and other veterinary interventions, promoting a more sustainable and eco-friendly approach to sheep farming. By integrating targeted nutritional strategies with improved health management, these findings provide valuable guidance for refining feeding and breeding practices to enhance overall livestock productivity and resilience.

This study offers new insights into the advantages of hybrid sheep in terms of immunity and metabolism, emphasizing their potential to enhance productivity and disease resistance in sheep farming. Our findings indicate that hybridization can improve immune responses and metabolic efficiency, both of which are critical for optimizing livestock performance. To translate these findings into practical applications, it is essential to consider strategies for selective breeding, dietary optimization, and management improvements.

The immune advantages observed in hybrid sheep indicate that crossbreeding strategies should prioritize combinations that enhance immune function. Our results indicate that certain hybrid groups have increased levels of immune-related metabolites and a more balanced rumen microbiota, which may contribute to improved disease resistance. Previous studies have explored candidate genes for high milk production in East Friesian sheep (Zhong et al., 2024) and high reproductive performance in Hu sheep (Li et al., 2022). We plan to investigate further the milk yield and lambing rates of DH sheep, as well as explore the hybrid advantages of DH sheep at the genetic level. Additionally, previous research demonstrated that Dorper×Chinese Mongolian crossbred sheep have better carcass traits but lower meat nutritional quality compared to Chinese Mongolian sheep, with these differences closely linked to rumen microbiota composition. This suggests that future studies could focus on investigating the relationship between meat nutritional quality and carcass traits.

Furthermore, numerous studies have shown that three-way hybridization significantly improves production efficiency (McClearn et al., 2020), making DH sheep an interesting and worthwhile candidate for use in three-way hybrid breeding programs, offering new approaches for practical production. Prior research has also shown that different sheep breeds possess distinct rumen microbiota compositions that influence growth, feed conversion efficiency, and fat deposition, which aligns with our findings (Cheng et al., 2022). Additionally, studies on Hu sheep, Charolais × Australian White × Hu sheep, and Charolais × Dorper × Hu sheep have demonstrated that hybridization can affect microbial community structure and characteristics to regulate metabolism and improve production performance (Wang et al., 2024). Future breeding programs should incorporate immune profiling and metabolic assessments to identify optimal hybrid combinations that maximize these benefits.

Future research should focus on identifying genetic markers associated with the superior immune function and metabolic traits observed in DH sheep, enabling the application of marker-assisted selection (MAS) to enhance precision breeding programs and ensure the stable inheritance of desirable traits. Additionally, large-scale field trials are crucial for validating the long-term benefits of Hu × EF crossbreeding under diverse environmental and management conditions. These studies will provide critical insights for refining breeding strategies and optimizing practical applications, ultimately contributing to the development of more resilient and high-performing livestock populations.

The enriched microbial profile of DH sheep, particularly the higher prevalence of Prevotella, contributes to improved fiber digestion and carbohydrate metabolism, thereby enhancing feed conversion efficiency and reducing overall resource consumption, including land and water use. Additionally, the distinctive rumen microbial composition, including a notable presence of *Methanobrevibacter*, indicates a potential shift in fermentation pathways that may lower methane (CH₄) emissions, thereby reducing the environmental footprint of ruminant farming. Incorporating precision nutrition strategies, such as probiotic supplementation or alternative feed additives, alongside comprehensive life cycle assessments (LCA), can further optimize these benefits. Moreover, the improved health and disease resistance observed in DH sheep may decrease the need for veterinary interventions, indirectly supporting environmental sustainability by minimizing antibiotic use and associated ecological impacts.

Our study highlights the significant differences in immune function, rumen microbiome composition, and metabolic profiles across sheep breeds, with DH sheep exhibiting superior immune responses and metabolic advantages. These differences are likely influenced by a combination of genetic and microbial factors, with crossbreeding playing a crucial role in enhancing disease resistance. Our findings provide valuable insights into the potential of crossbreeding to improve livestock health and productivity. Future studies should focus on further elucidating the mechanisms underlying these differences, particularly how bile acids and specific microbial taxa influence immune responses and overall health.

## 8 Conclusion

DH sheep exhibited enhanced immune function, characterized by elevated lymphocyte counts and increased serum IgA levels, potentially enhancing disease resistance. The rumen microbiome of DH sheep was significantly enriched in *Prevotellaceae* (e.g., *Prevotella* spp.), a group associated with fiber degradation and host-microbial co-metabolism. Additionally, the concentrations of CDCA and 3*β*-HDCA were higher in DH sheep, supporting their roles as biomarkers for bile acid homeostasis and metabolic efficiency. These metabolic advantages included enhanced carbohydrate fermentation pathways (e.g., KEGG pathways of carbohydrate metabolism) and xenobiotic degradation, likely mediated by the enriched presence of *Methanobrevibacter* and other syntrophic microbes. Compared to Hu and EF sheep, DH sheep exhibited better energy utilization efficiency and environmental adaptability, potentially due to the inheritance of high carbohydrate enzyme activity from Hu sheep and compensatory metabolic traits from EF sheep. Strong positive correlations between rumen microbiota and bile acids, along with upregulated immune responses, indicate improved bile acid metabolism and immunomicrobial crosstalk, likely driven by heterosis. In contrast, EF and Hu sheep showed more regulatory or negative correlations, highlighting breed-specific differences in immune indicator–microbiota interactions. Overall, this study reveals that crossbreeding optimizes immune and metabolic traits by integrating beneficial microbiome functions and host genetic backgrounds.

## Data Availability

The datasets supporting the conclusions of this article are available in the NCBI Sequence Read Archive (SRA) repository under accession numbers PRJNA1127428 and PRJNA1234541. The metabolome data are available in the NGDC database under accession number PRJCA027368.

## Footnotes

^1^ https://github.com/OpenGene/fastp

^2^ http://ccb.Jhu.edu/software/FLASH/

^3^ https://github.com/torognes/vsearch

^4^ http://www.metware.cn/

## Glossary

Hu: Hu sheep
EF: East Friesian sheep
DH: East Friesian*Hu Crossbred sheep
VFAs: Volatile fatty acids
SCFAs: Short-chain fatty acids
WBC: White blood cell
LYM: lymphocyte count
LYM%: Percentage of lymphocytes
ALY: Abnormal lymph count
IgM: Immunoglobulin M
IgA: Immunoglobulin A
IgG: immunoglobulin G
CD4: Cluster of differentiation 4
CD8: Cluster of differentiation 8
OTUs: Operational taxonomic units
PCoA: Principal co-ordinates analysis
HCA: Hierarchical cluster analysis
QC: Quality control
RT: Retention time
PCA: Principle component analysis
OPLS-DA: Orthogonal partial least squares discriminate analysis
VIP: Variable importance in the projection
A/P: Acetate to propionate ratio
TIC: Total ion chromatogram
6-ketoLCA: 5-β-Cholanic Acid-3α-ol-6-one
CDCA: Chenodeoxycholic acid
3β-HDCA: 3β-dehydrocholic acid
