# Supplementary material for: Discrepancies in the rumen microbiome, metabolome, and serum metabolome among Hu sheep, East Friesian sheep, and East Friesian × Hu crossbred sheep
Source: Front Microbiol. 2025 Apr 28;16:1498050. doi: 10.3389/fmicb.2025.1498050 (PMC12066648; doi:10.3389/fmicb.2025.1498050)

**Supplementary information**

Including **7 Tables** and **13 figures**

**Table S1** Rumen microbial composition and relative abundances of Hu, EF, and DH sheep (phylum level)

| Taxonomy | DH(n=6) | Hu(n=6) | EF(n=6) | SEM | P-value | FDR |
| --- | --- | --- | --- | --- | --- | --- |
| p__Euryarchaeota | 0.022318742 | 0.008912455 | 0.002977086 | 0.009908387 | 0.024109575 | 0.160730503 |
| p__Synergistota | 2.50702E-05 | 0.000401123 | 0.000156689 | 0.000190826 | 0.011705334 | 0.117053342 |
| Others | 0.00314631 | 0.003954824 | 0.010880465 | 0.004251184 | 0.010896826 | 0.117053342 |

**Table S2** Rumen microbial composition and relative abundances of Hu, EF, and DH sheep (order level)

| Taxonomy | DH(n=6) | Hu(n=6) | EF(n=6) | SEM | P-value | FDR |
| --- | --- | --- | --- | --- | --- | --- |
| o__Methanobacteriales | 0.022318742 | 0.008912455 | 0.002977086 | 0.009908387 | 0.024109575 | 0.256297962 |
| o__Acidaminococcales | 0.009608153 | 0.109845066 | 0.02493231 | 0.053994513 | 0.034243141 | 0.256297962 |
| o__Rhizobiales | 2.50702E-05 | 0 | 0 | 1.44743E-05 | 0.034218118 | 0.256297962 |
| o__unidentified_Alphaproteobacteria | 8.14781E-05 | 0 | 0 | 4.70414E-05 | 0.034501649 | 0.256297962 |
| o__Enterobacterales | 0.000883724 | 0.003597573 | 0.028824458 | 0.015408035 | 0.027761416 | 0.256297962 |
| o__Synergistales | 2.50702E-05 | 0.000401123 | 0.000156689 | 0.000190826 | 0.011705334 | 0.256297962 |
| o__Campylobacterales | 0 | 2.50702E-05 | 0.000213097 | 0.000116471 | 0.006690377 | 0.256297962 |

**Table S3** Rumen microbial composition and relative abundances of Hu, EF, and DH sheep (class level)

| Taxonomy | DH(n=6) | Hu(n=6) | EF(n=6) | SEM | P-value | FDR |
| --- | --- | --- | --- | --- | --- | --- |
| c__Methanobacteria | 0.022318742 | 0.008912455 | 0.002977086 | 0.009908387 | 0.024109575 | 0.130191707 |
| c__Negativicutes | 0.030698456 | 0.211542318 | 0.092941486 | 0.091873876 | 0.003479688 | 0.090320093 |
| c__Alphaproteobacteria | 0.00035725 | 0.000150421 | 5.64079E-05 | 0.000153906 | 0.021762436 | 0.130191707 |
| c__Synergistia | 2.50702E-05 | 0.000401123 | 0.000156689 | 0.000190826 | 0.011705334 | 0.105348008 |
| c__Campylobacterota | 0 | 2.50702E-05 | 0.000213097 | 0.000116471 | 0.006690377 | 0.090320093 |

**Table S4** Rumen microbial composition and relative abundances of Hu, EF, and DH sheep (family level)

| Taxonomy | DH(n=6) | Hu(n=6) | EF(n=6) | SEM | P-value | FDR |
| --- | --- | --- | --- | --- | --- | --- |
| f__Methanobacteriaceae | 0.022318742 | 0.008912455 | 0.002977086 | 0.009908387 | 0.024109575 | 0.303347117 |
| f__Rikenellaceae | 0.034233353 | 0.031475632 | 0.005590654 | 0.015801062 | 0.034243141 | 0.303347117 |
| f__Streptococcaceae | 0.10740699 | 1.25351E-05 | 0.000244434 | 0.061937382 | 0.036269764 | 0.303347117 |
| f__Lachnospiraceae | 0.097698556 | 0.083696851 | 0.193654733 | 0.059853114 | 0.02468019 | 0.303347117 |
| f__Acidaminococcaceae | 0.009608153 | 0.109845066 | 0.02493231 | 0.053994513 | 0.034243141 | 0.303347117 |
| f__Selenomonadaceae | 0.020971219 | 0.090515945 | 0.016891045 | 0.041379831 | 0.049787068 | 0.381700857 |
| f__Veillonellaceae | 0 | 0.01081779 | 0.050823556 | 0.026772249 | 0.004226288 | 0.303347117 |
| f__Beijerinckiaceae | 2.50702E-05 | 0 | 0 | 1.44743E-05 | 0.034218118 | 0.303347117 |
| f__Neisseriaceae | 1.88026E-05 | 1.25351E-05 | 0.000250702 | 0.000135733 | 0.015948241 | 0.303347117 |
| f__Pasteurellaceae | 1.88026E-05 | 1.88026E-05 | 0.000150421 | 7.599E-05 | 0.026610359 | 0.303347117 |
| f__Synergistaceae | 2.50702E-05 | 0.000401123 | 0.000156689 | 0.000190826 | 0.011705334 | 0.303347117 |
| f__Campylobacteraceae | 0 | 2.50702E-05 | 0.000213097 | 0.000116471 | 0.006690377 | 0.303347117 |

**Table S5** Rumen microbial composition and relative abundances of Hu, EF, and DH sheep (genus level)

| Taxonomy | DH(n=6) | Hu(n=6) | EF(n=6) | SEM | P-value | FDR |
| --- | --- | --- | --- | --- | --- | --- |
| *g__Methanobrevibacter* | 0.01526775 | 0.008674288 | 0.00217484 | 0.006546511 | 0.03804423 | 0.226569436 |
| *g__Methanosphaera* | 0.007019655 | 0.000219364 | 0.000789711 | 0.003772299 | 0.005969345 | 0.110794909 |
| *g__Asteroleplasma* | 1.88026E-05 | 0.000144154 | 0.056758925 | 0.032722799 | 0.005020288 | 0.110794909 |
| *g__Streptococcus* | 0.10740699 | 1.25351E-05 | 0.000244434 | 0.061937382 | 0.036269764 | 0.226569436 |
| *g__Eubacterium* | 0.000990273 | 6.8943E-05 | 9.40132E-05 | 0.000524843 | 0.033720737 | 0.226569436 |
| *g__Pseudoramibacter* | 0 | 0.00038232 | 0.000338448 | 0.000209221 | 0.02026702 | 0.1778994 |
| *g__Acetitomaculum* | 0.019134828 | 0.025446249 | 0.00222498 | 0.012006973 | 0.014243395 | 0.140653526 |
| *g__Agathobacter* | 0 | 0.001754914 | 0.000112816 | 0.000982254 | 0.036635661 | 0.226569436 |
| *g__Blautia* | 0.000250702 | 0 | 0.000607952 | 0.000305528 | 0.025085874 | 0.208608844 |
| *g__Coprococcus* | 0.00033218 | 0.000106548 | 6.26755E-06 | 0.000166926 | 0.031926695 | 0.226569436 |
| *g__Dorea* | 0 | 6.26755E-06 | 0.000407391 | 0.000233419 | 0.000598014 | 0.047243131 |
| *g__Lachnospira* | 6.26755E-06 | 0.000181759 | 0.01943567 | 0.011167255 | 0.000428587 | 0.047243131 |
| *g__Moryella* | 0.001266045 | 0.000388588 | 0 | 0.000648563 | 0.009116037 | 0.110794909 |
| *g__Oribacterium* | 0.004738267 | 0.000319645 | 0.000206829 | 0.002584276 | 0.006637827 | 0.110794909 |
| *g__Roseburia* | 0 | 0.000112816 | 0.013744735 | 0.007903161 | 0.008755437 | 0.110794909 |
| *g__Syntrophococcus* | 0.001855195 | 0.003459687 | 0.016182812 | 0.007849978 | 0.038717562 | 0.226569436 |
| *g__Oscillibacter* | 0 | 6.26755E-06 | 0.000119083 | 6.70169E-05 | 0.004783323 | 0.110794909 |
| *g__Paludicola* | 0 | 0 | 0.000175491 | 0.00010132 | 0.008542975 | 0.110794909 |
| *g__unidentified_Ruminococcaceae* | 0 | 6.26755E-06 | 0.000564079 | 0.000323877 | 0.004075319 | 0.110794909 |
| *g__Butyricicoccus* | 0 | 0 | 0.000394856 | 0.00022797 | 0.034501649 | 0.226569436 |
| *g__Monoglobus* | 0.000463799 | 0.000338448 | 0.000162956 | 0.000151116 | 0.042963444 | 0.242436578 |
| *g__Acidaminococcus* | 0 | 0.005383825 | 0.006543321 | 0.003491539 | 0.010540373 | 0.113329747 |
| *g__Mitsuokella* | 0 | 0.004111512 | 0.001667168 | 0.002067962 | 0.015980287 | 0.148522669 |
| *g__Dialister* | 0 | 0.003240323 | 0.048504563 | 0.027117165 | 0.008072318 | 0.110794909 |
| *g__Megasphaera* | 0 | 0.007514791 | 0.002043221 | 0.003885549 | 0.010759153 | 0.113329747 |
| *g__Bibersteinia* | 0 | 0 | 2.50702E-05 | 1.44743E-05 | 0.034218118 | 0.226569436 |
| *g__Pyramidobacter* | 0 | 0.000338448 | 0.000150421 | 0.000169572 | 0.003122158 | 0.110794909 |
| *g__Campylobacter* | 0 | 2.50702E-05 | 0.000213097 | 0.000116471 | 0.006690377 | 0.110794909 |

**Table S6** Rumen microbial composition and relative abundances of Hu, EF, and DH sheep (species level)

| Taxonomy | DH(n=6) | Hu(n=6) | EF(n=6) | SEM | P-value | FDR |
| --- | --- | --- | --- | --- | --- | --- |
| *g__unidentified_Prevotellaceae;s__metagenome* | 0 | 0.001197102 | 0.006236211 | 0.003309482 | 0.007788347 | 0.273375216 |
| *g__Asteroleplasma;s__gut_metagenome* | 1.88026E-05 | 0.000137886 | 0.05674639 | 0.032717366 | 0.004470022 | 0.273375216 |
| *s__Pseudoramibacter_sp* | 0 | 0.000369785 | 0.00030711 | 0.0001979 | 0.02026702 | 0.405340406 |
| *s__Clostridium_sp_SY8519* | 0 | 5.64079E-05 | 0.000658093 | 0.000364758 | 0.01574545 | 0.359896009 |
| *s__Eubacterium_ruminantium* | 0 | 0.000231899 | 0 | 0.000133887 | 0.008542975 | 0.273375216 |
| *g__unidentified_Lachnospiraceae;s__human_gut_metagenome* | 0 | 4.38728E-05 | 0.002588498 | 0.001481967 | 0.044003154 | 0.445914474 |
| *s__Ruminococcus_sp* | 0.045916065 | 0.008479994 | 0 | 0.024432406 | 0.044680909 | 0.445914474 |
| *s__Ruminococcus_sp_A254_MGS_108* | 0 | 6.26755E-06 | 0.000106548 | 5.97886E-05 | 0.024869266 | 0.442120293 |
| *s__Acidaminococcus_fermentans* | 0 | 9.40132E-05 | 0.000137886 | 7.04461E-05 | 0.035053017 | 0.445914474 |
| *s__Dialister_succinatiphilus* | 0 | 0.002137234 | 0.048385479 | 0.027339296 | 0.005679245 | 0.273375216 |
| *s__Megasphaera_elsdenii* | 0 | 0.00414285 | 0.000451264 | 0.002272834 | 0.011698047 | 0.311947933 |
| *s__Megasphaera_hexanoica* | 0 | 0.000557812 | 0.001115624 | 0.000557812 | 0.031888278 | 0.445914474 |
| *s__Campylobacter_fetus* | 0 | 1.88026E-05 | 0.000194294 | 0.000107161 | 0.006964932 | 0.273375216 |

**Table S7** Metagenomic sequencing data statistics table\

| **Sample** | **bases(G)** | **Q20** | **Q30** | **GC** |
| --- | --- | --- | --- | --- |
| Hu.8 | 7.95 | 98.98 | 96.56 | 40.2 |
| Hu.9 | 9.25 | 98.87 | 96.21 | 39.62 |
| Hu.10 | 7.13 | 98.88 | 96.19 | 48 |
| Hu.11 | 9.28 | 99 | 96.56 | 46.83 |
| Hu.12 | 7.34 | 98.97 | 96.53 | 43.62 |
| Hu.13 | 7.61 | 99 | 96.53 | 46.86 |
| EF.7 | 8.94 | 98.94 | 96.4 | 49.09 |
| EF.8 | 9.08 | 99.05 | 96.72 | 44 |
| EF.9 | 8.76 | 99.06 | 96.74 | 45.28 |
| EF.10 | 8.17 | 98.98 | 96.5 | 45.77 |
| EF.11 | 8.97 | 99.05 | 96.74 | 46.02 |
| EF.12 | 7.67 | 98.97 | 96.5 | 48.84 |
| DH.1 | 7.98 | 98.97 | 96.48 | 50.8 |
| DH.2 | 8.24 | 98.85 | 96.15 | 51.81 |
| DH.3 | 7.51 | 98.83 | 96.04 | 49.88 |
| DH.4 | 9.26 | 98.95 | 96.41 | 51.05 |
| DH.5 | 11.27 | 98.94 | 96.41 | 50.72 |
| DH.6 | 8 | 98.96 | 96.44 | 45.83 |


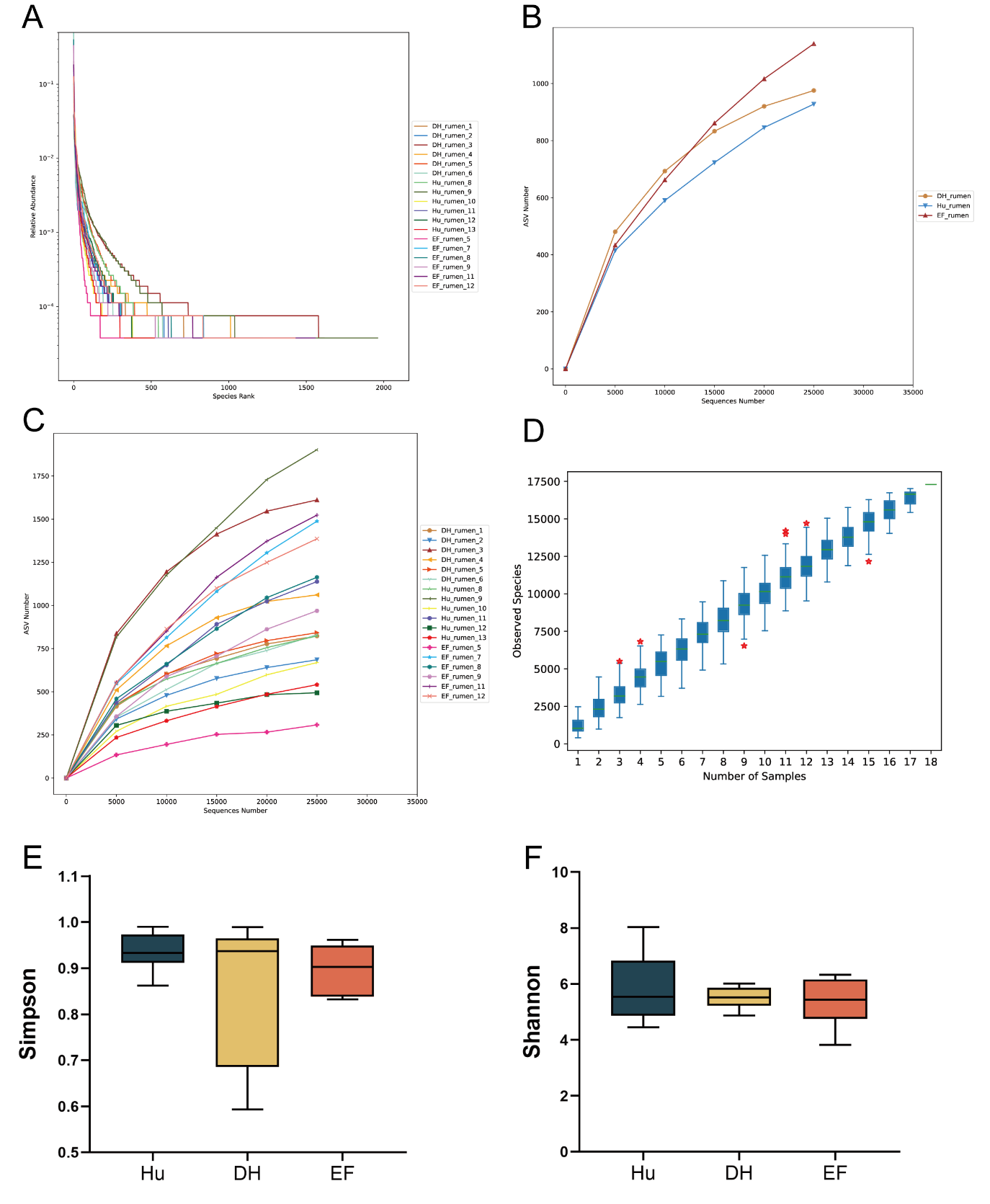
**Figure S1 (A)** Rank Abundance curve of each sample based on ASV. **(B)** Group dilution curve based on ASV. **(C)** Dilution curve of each sample based on ASV. **(D)** Box diagram of species accumulation based on ASV. **(E)** Simpson index. **(F)** Shannon index.

**Figure S2** Visual presentation of species annotation results. KRONA was used to visualize the species annotation results. In the display results, the circles from the inside to the outside represent different classification levels, and the size of the fan represents the relative proportion of different ASV annotation results.


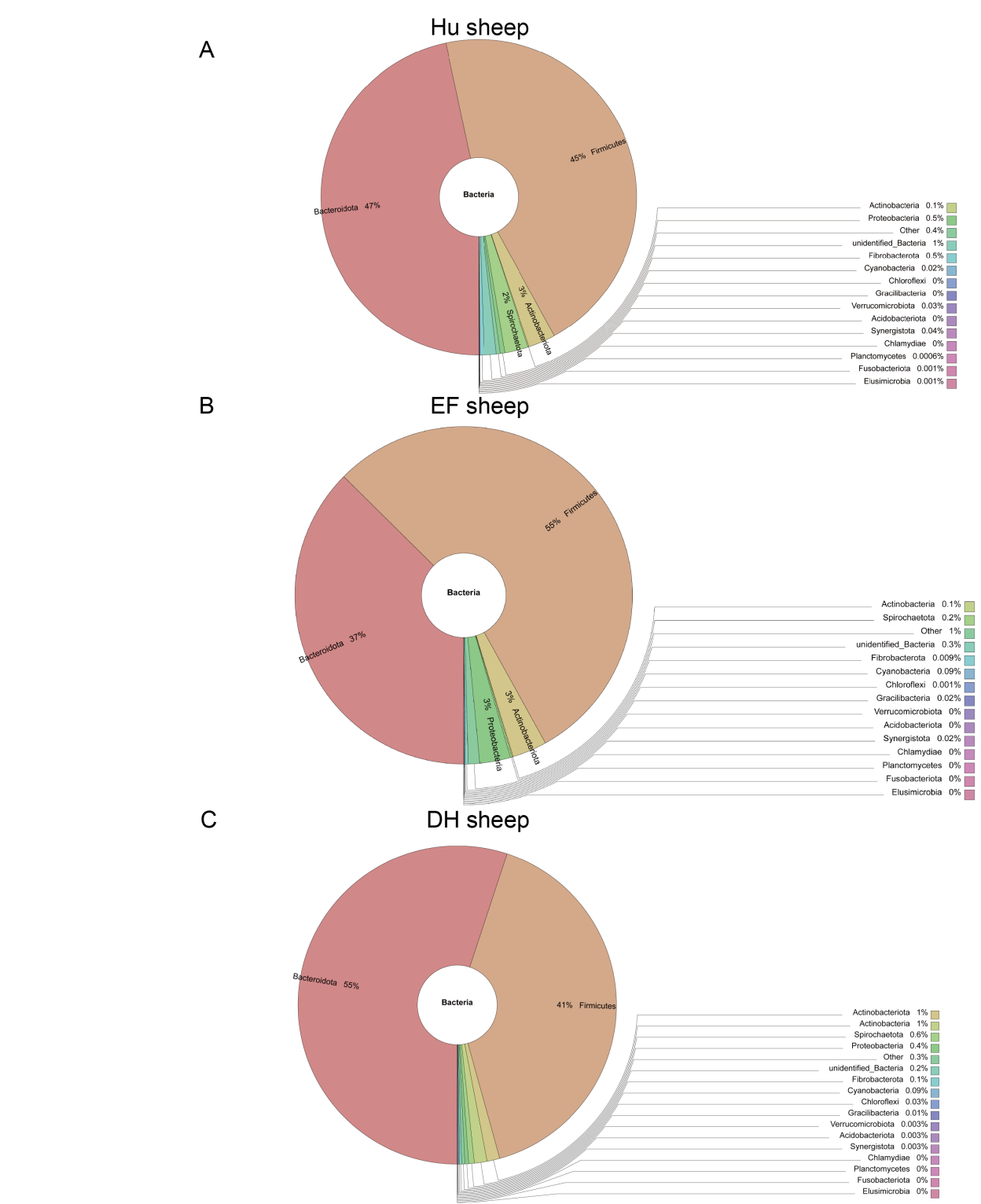


**Figure S3** The composition of different microorganisms in rumen contents samples among Hu, EF, DH sheep. **(A)** Based on the detected ASVs petal pattern **(B)** Stack map of species abundance at Phylum level. Comparison of Hu sheep and DH sheep and EF sheep rumen microbiota was performed using Wilcoxon rank sum test for significantly different domains at family**(C)**, genus**(D)**, plylum**(E)**, class**(F)** and order level**(G)**. * *p* <0.05, ** *p* <0.01


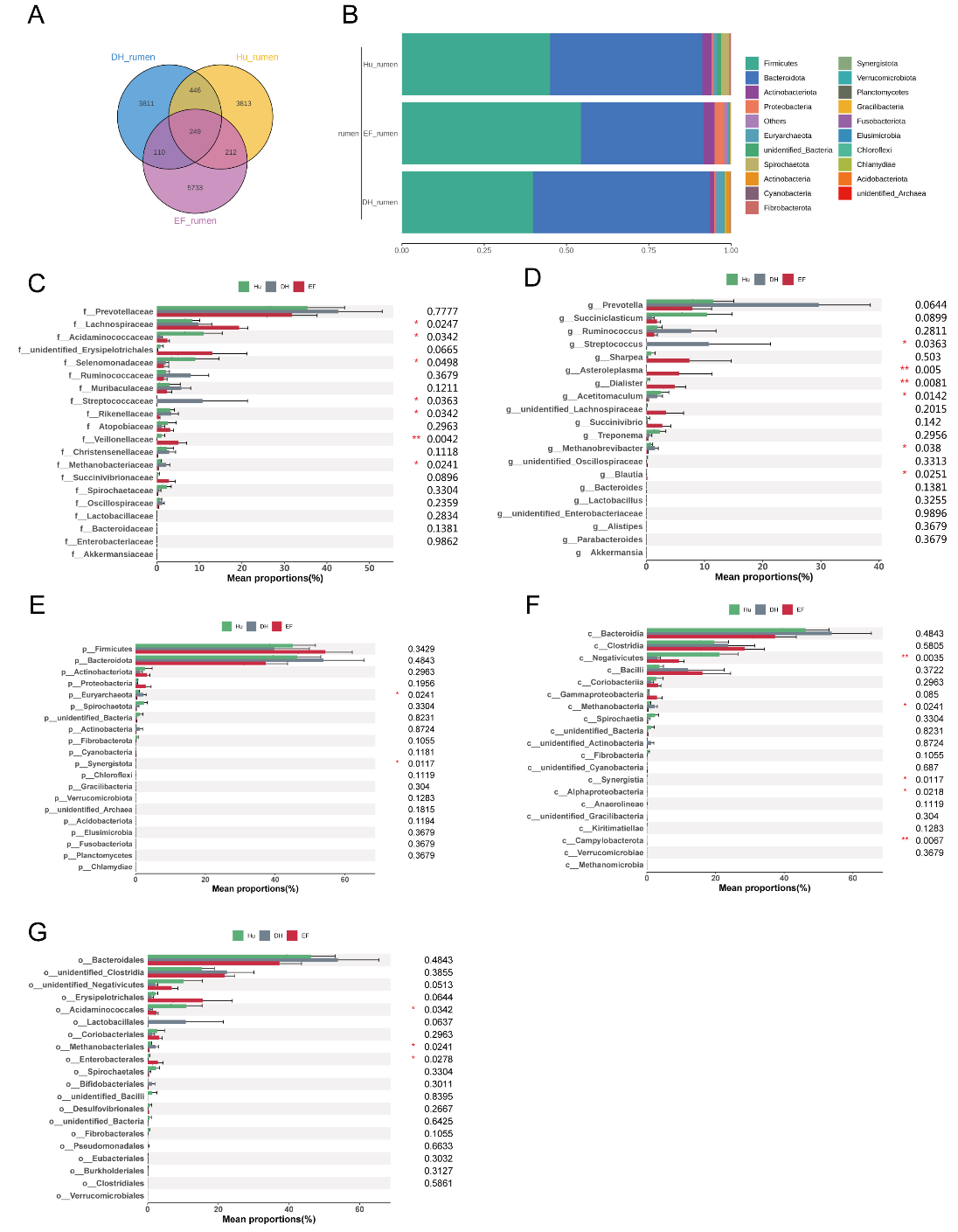


**Figure S4** Significantly different bacterial taxa in the rumen microbiome of Hu, DH, and EF sheep based on metagenomic sequencing. (A) ACE index of alpha diversity. (B) Chao1 index of alpha diversity. (C) PCoA and (D) NMDS analysis of the rumen microbiome at the species level based on metagenomic sequencing. (E) LEfSe analysis of the rumen microbiome in the three groups. Significant differences were identified using LEfSe analysis, with an LDA score > 4 and p < 0.05. Red indicates taxa enriched in DH sheep, green indicates taxa enriched in EF sheep, and blue indicates taxa enriched in Hu sheep. (F–I) Stacked bar plots of species abundance in individual samples (N = 6 per group) at the phylum (F), family (G), genus (H), and species (I) levels.

**
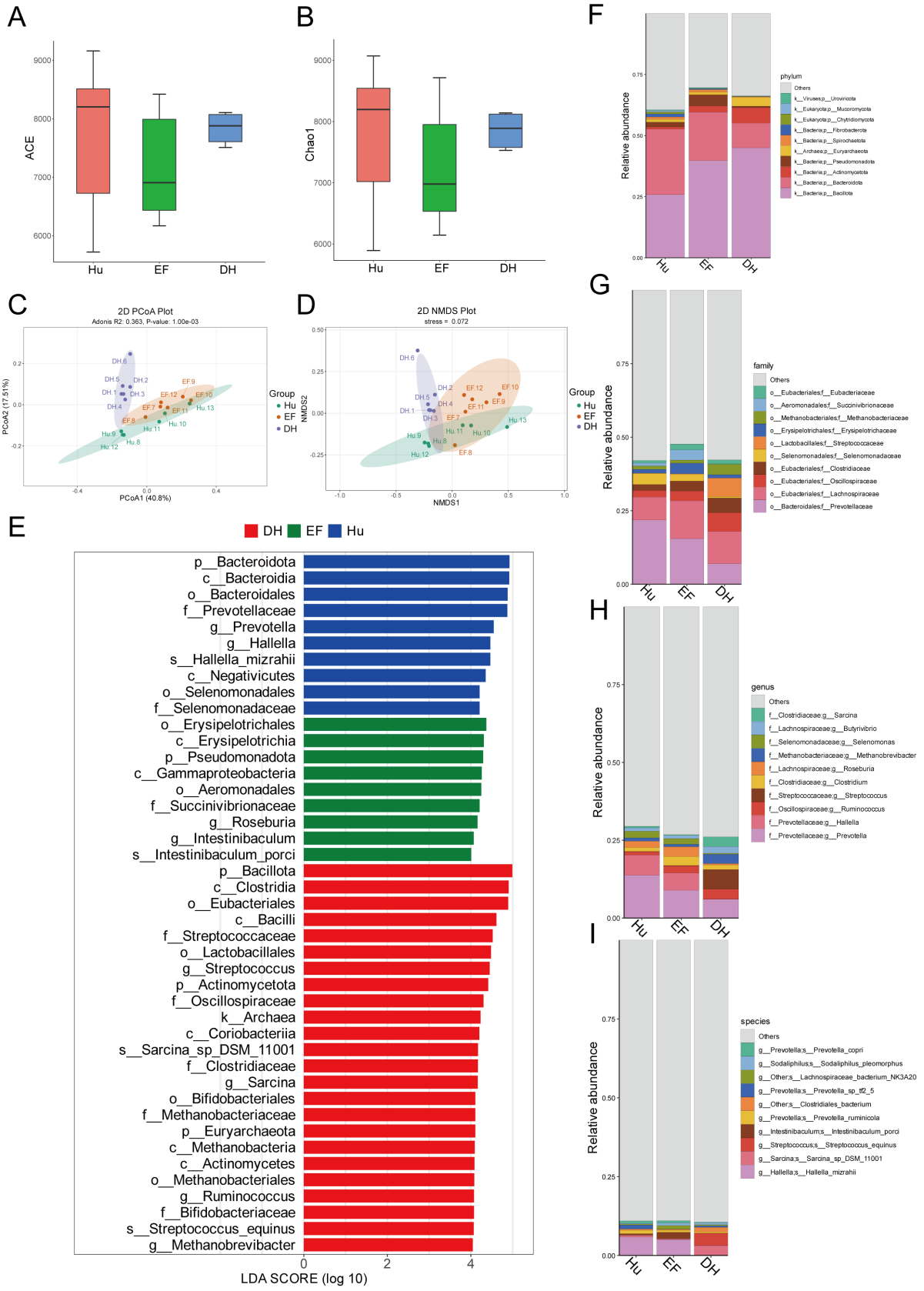
**

**Figure S5** The total ion chromatograms (TIC) plot of quality control (QC) samples of rumen metabolites in A positive ion mode and B negative ion mode.


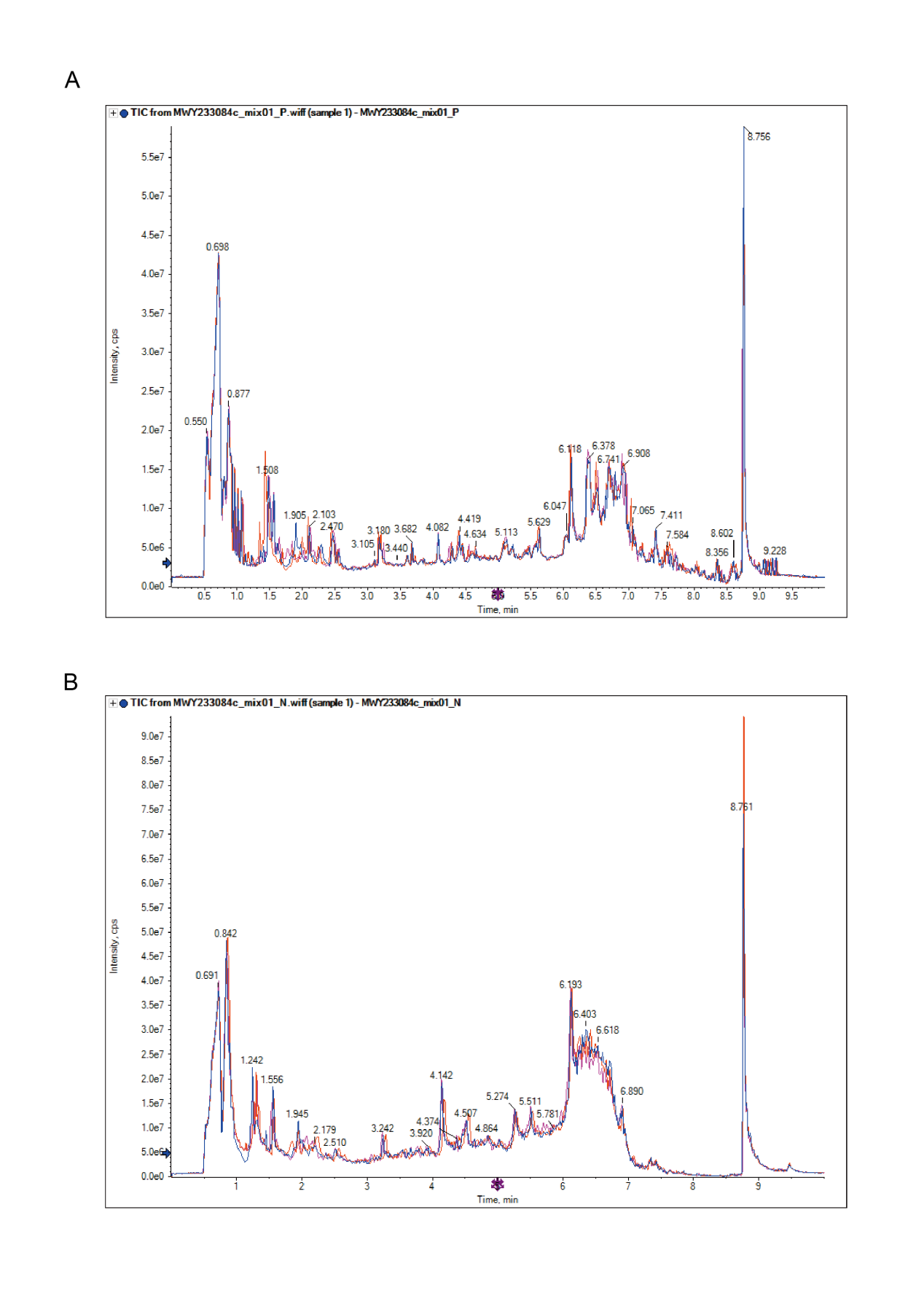


**Figure S6** Orthogonal partial least squares discriminant analysis (OPLS-DA) **(A, C, E)** and response permutation testing (RPT) **(B, D, F)** of rumen metabolites between Hu, EF, and DH sheep. R^2^X and R^2^Y represent the interpretation rate of the built model to the X and Y matrix, R^2^X (cum) and R^2^Y (cum) represent the cumulative interpretation rate; Q^2^ indicates the predictive power of the model


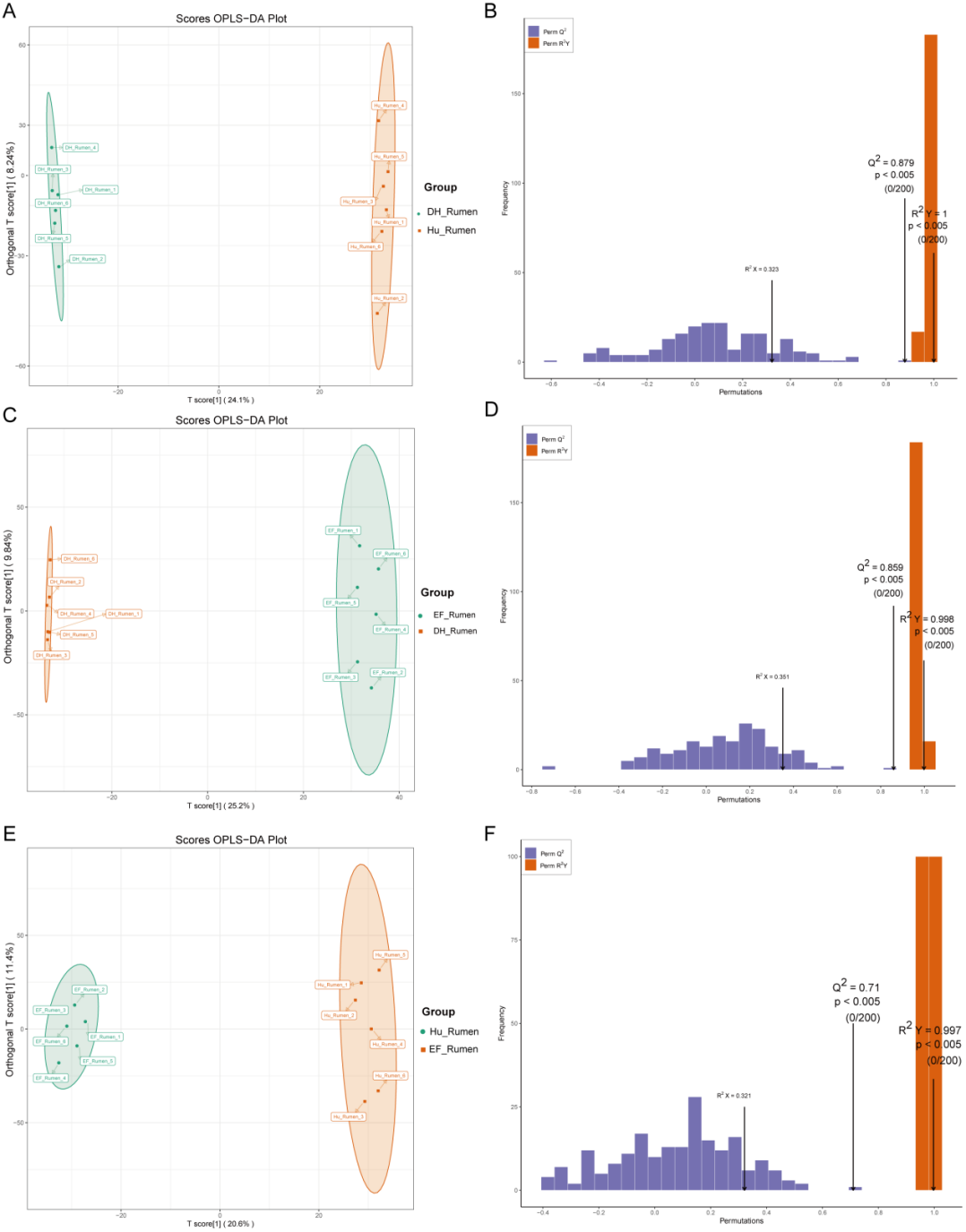


**Figure S7 (A)** The pie diagrams contain the numbers and percentages of identified metabolites classes in the rumen contents of Hu sheep and DH sheep and EF sheep. (**B&C)** A Volcano plot of differential rumial metabolites of DH sheep vs. EF sheep and DH sheep vs. Hu sheep. (**D&E)** Clustering heat map of differential rumial metabolites of DH sheep vs. Hu sheep and DH sheep vs. EF sheep

**
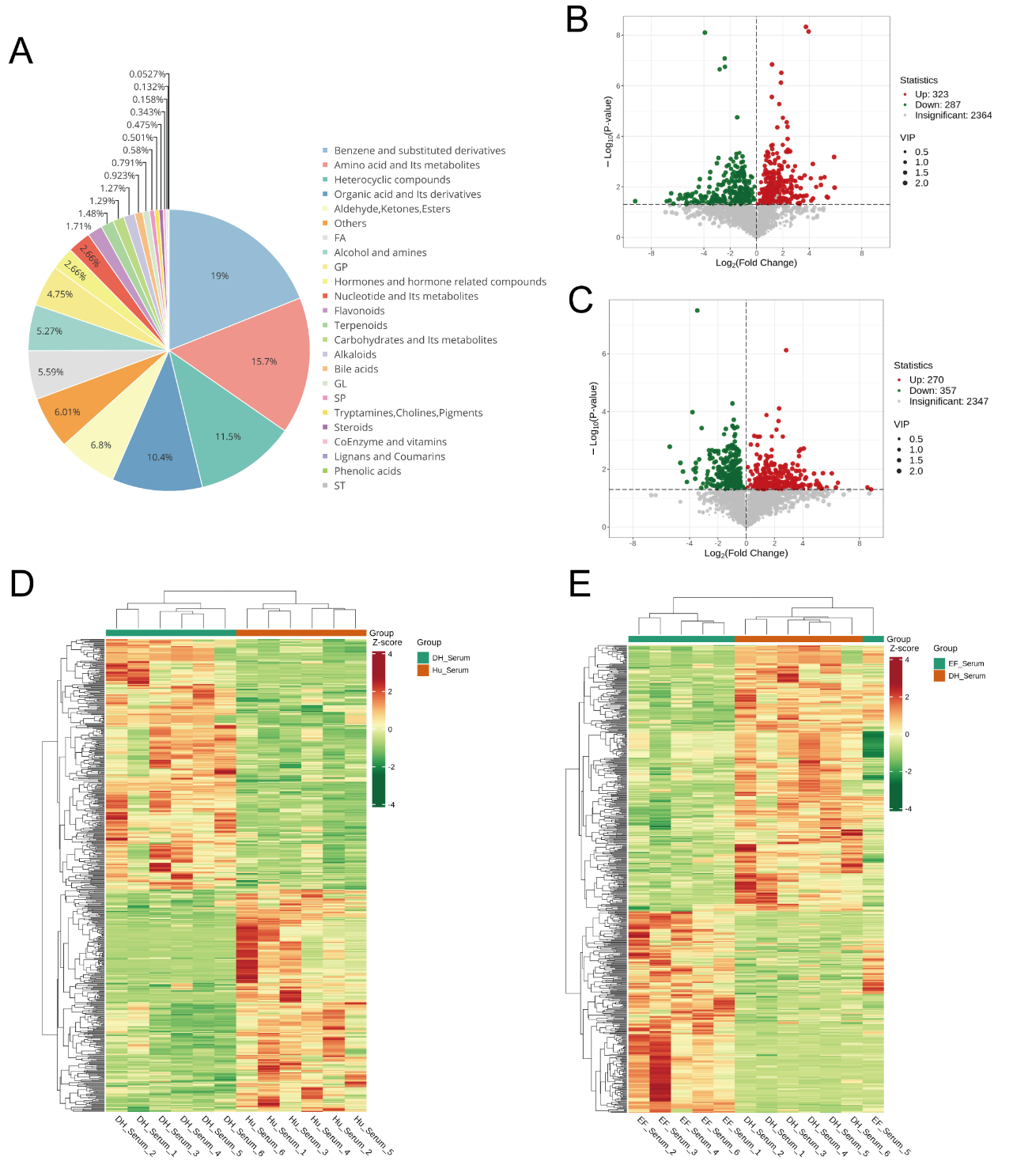
**

**Figure S8** Bile acid-targeted metabolic violin plots. All selected plots had bile acid *p* values less than 0.05.

**
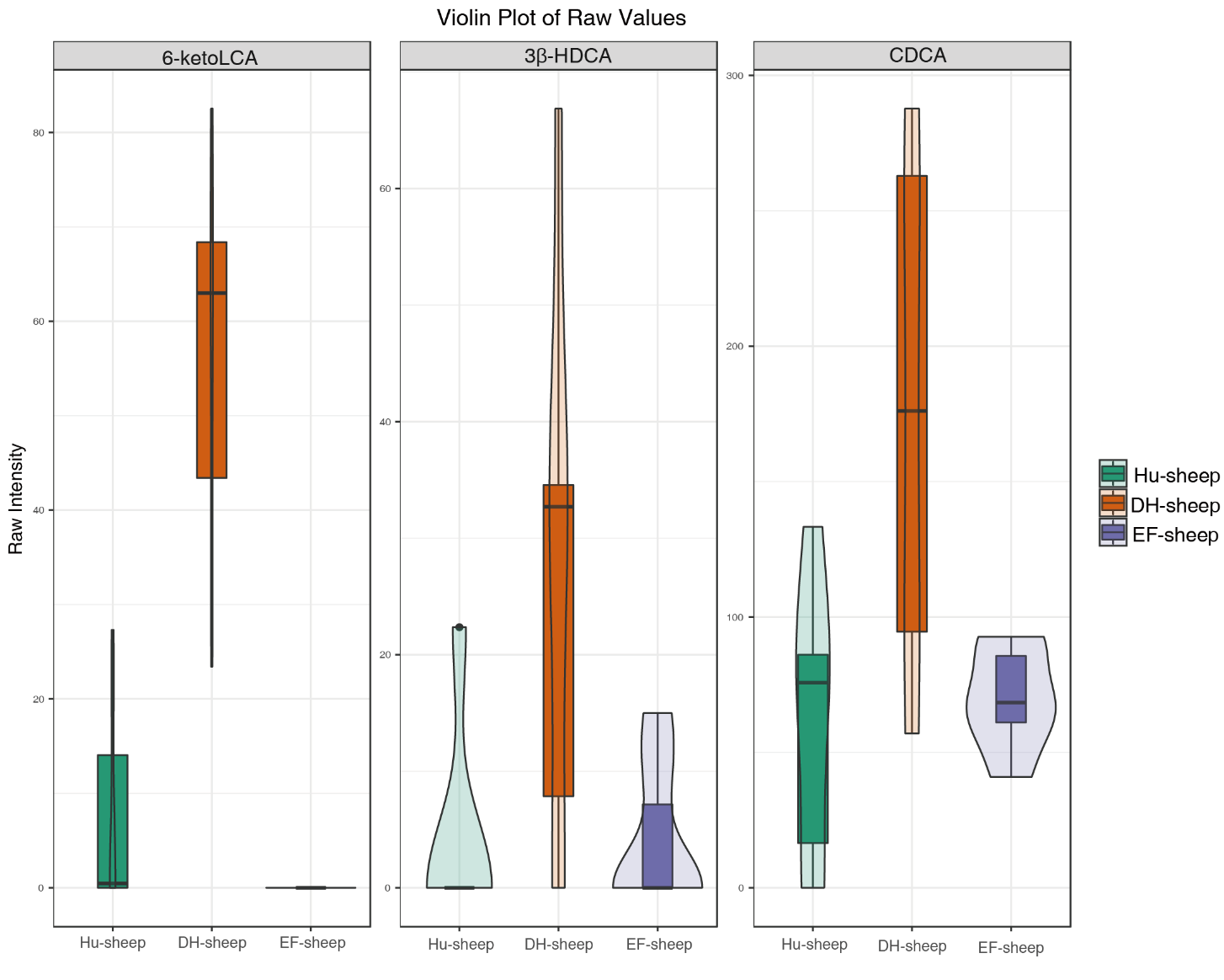
**

**Figure S9** VIP score analysis for differential non-esterified fatty acid metabolites in rumen. Non-esterified fatty acid with VIP scores >1 were selected and ranked based on VIP scores. The length of the bar indicates the value of the contribution of this metabolite to the difference between the two groups. The colour of the bar indicates the significance of the difference between the two groups of samples. * indicates *P*<0.05, ** indicates *P*<0.01 and *** indicates *P*<0.001.


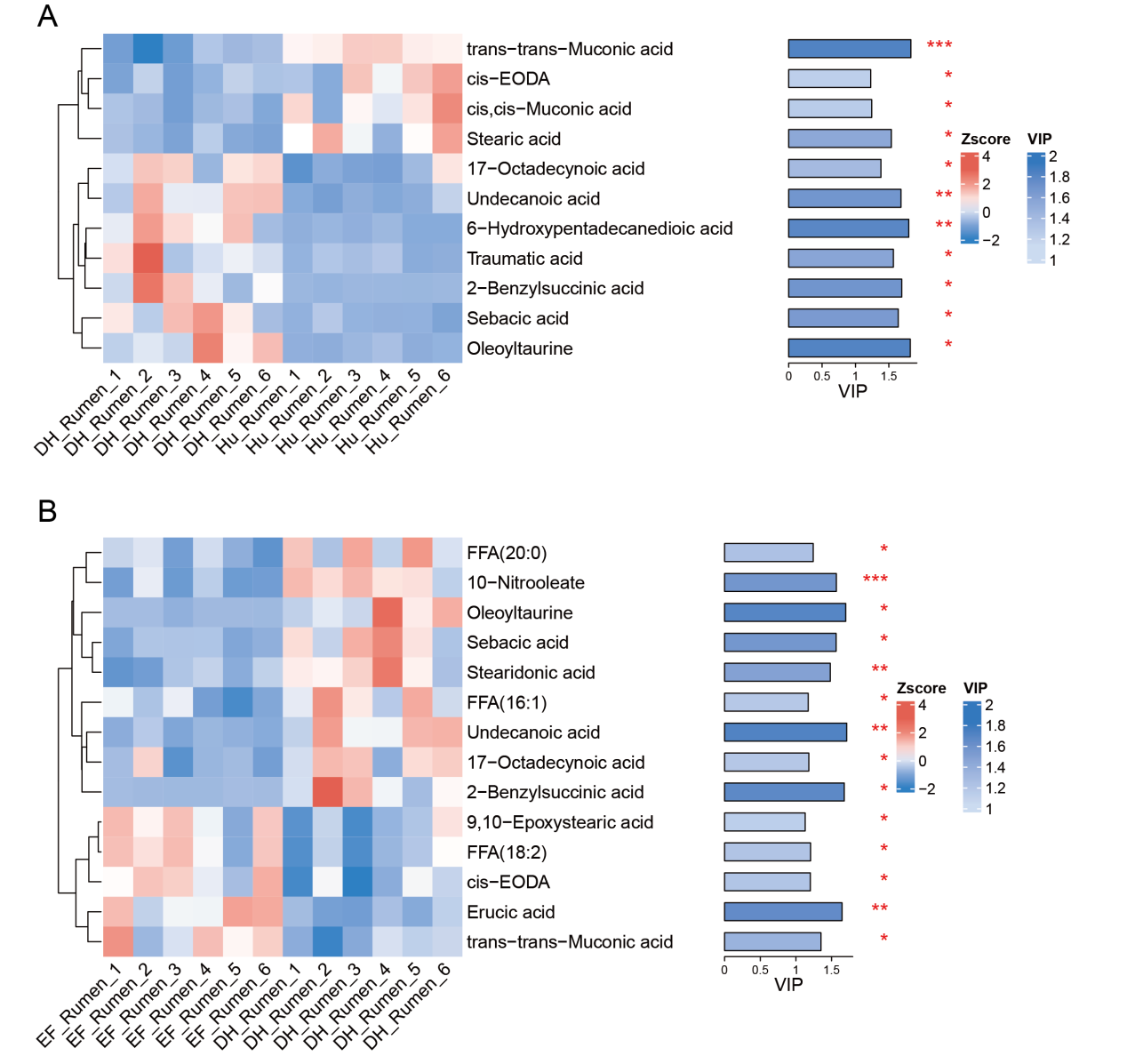


**Figure S10** The total ion chromatograms (TIC) plot of quality control (QC) samples of serum metabolites in A positive ion mode and B negative ion mode**(A&B)**


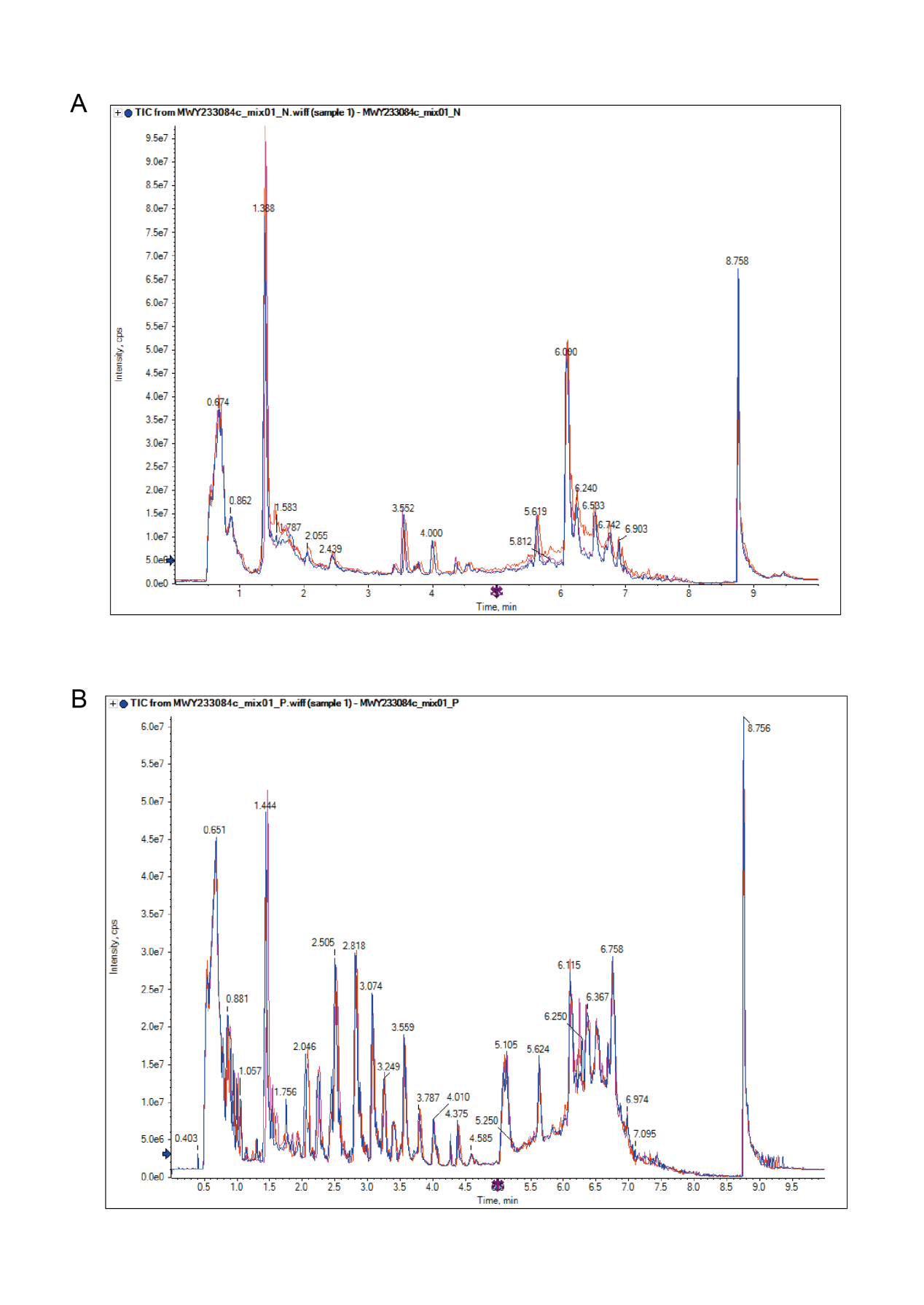


**Figure S11** Orthogonal partial least squares discriminant analysis (OPLS-DA) **(A, C, E)** and response permutation testing (RPT) **(B, D, F)** of serum metabolites between Hu, EF, and DH sheep. R^2^X and R^2^Y represent the interpretation rate of the built model to the X and Y matrix, R^2^X (cum) and R^2^Y (cum) represent the cumulative interpretation rate; Q^2^ indicates the predictive power of the model


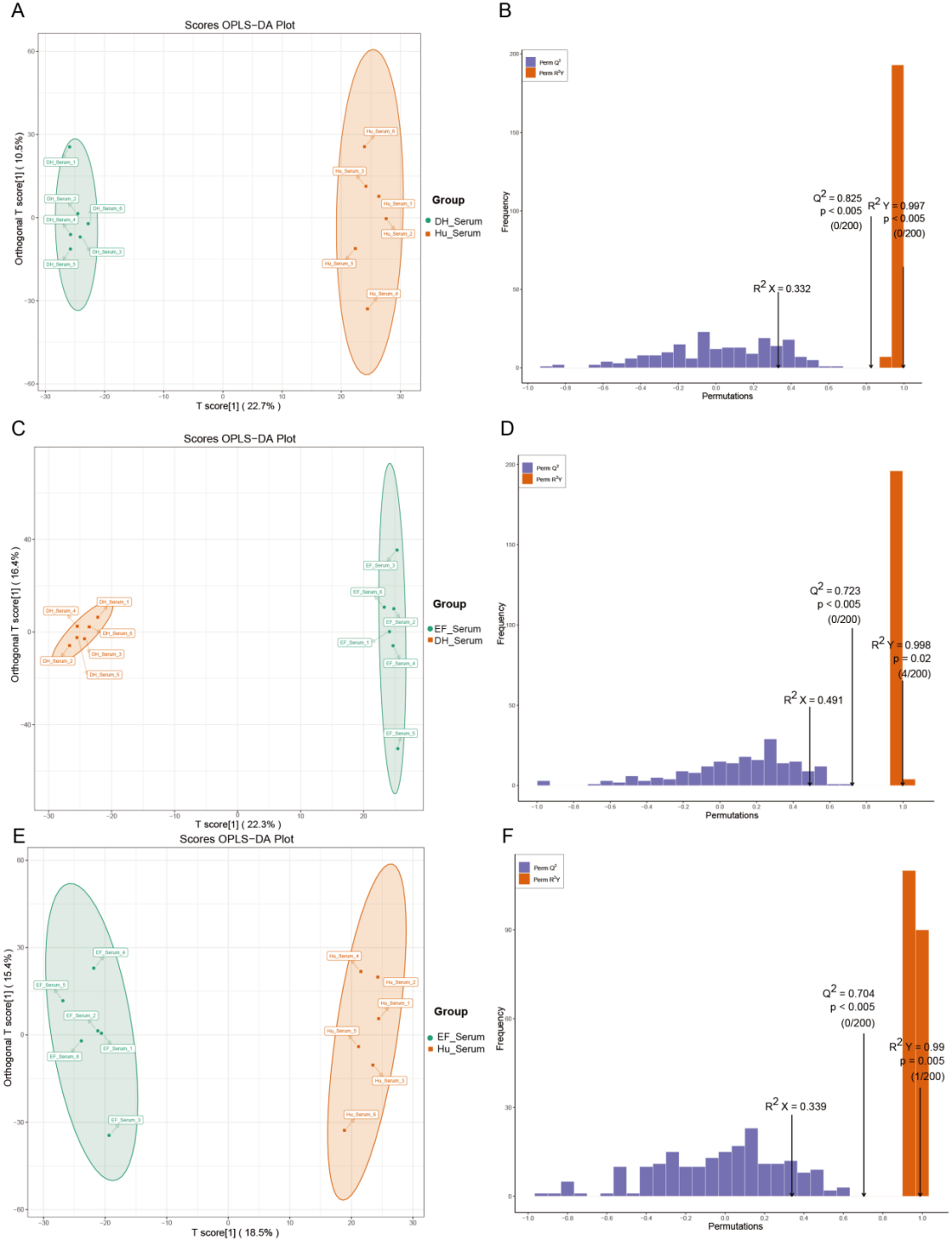


**Figure S12 (A)** The pie diagrams contain the numbers and percentages of identified metabolites classes in the serum of Hu sheep and DH sheep and EF sheep. **(B&C)** A Volcano plot of differential serum metabolites of DH sheep vs. EF sheep and DH sheep vs. Hu sheep. **(D&E)** Clustering heat map of differential serum metabolites of DH sheep vs. Hu sheep and DH sheep vs. EF sheep.


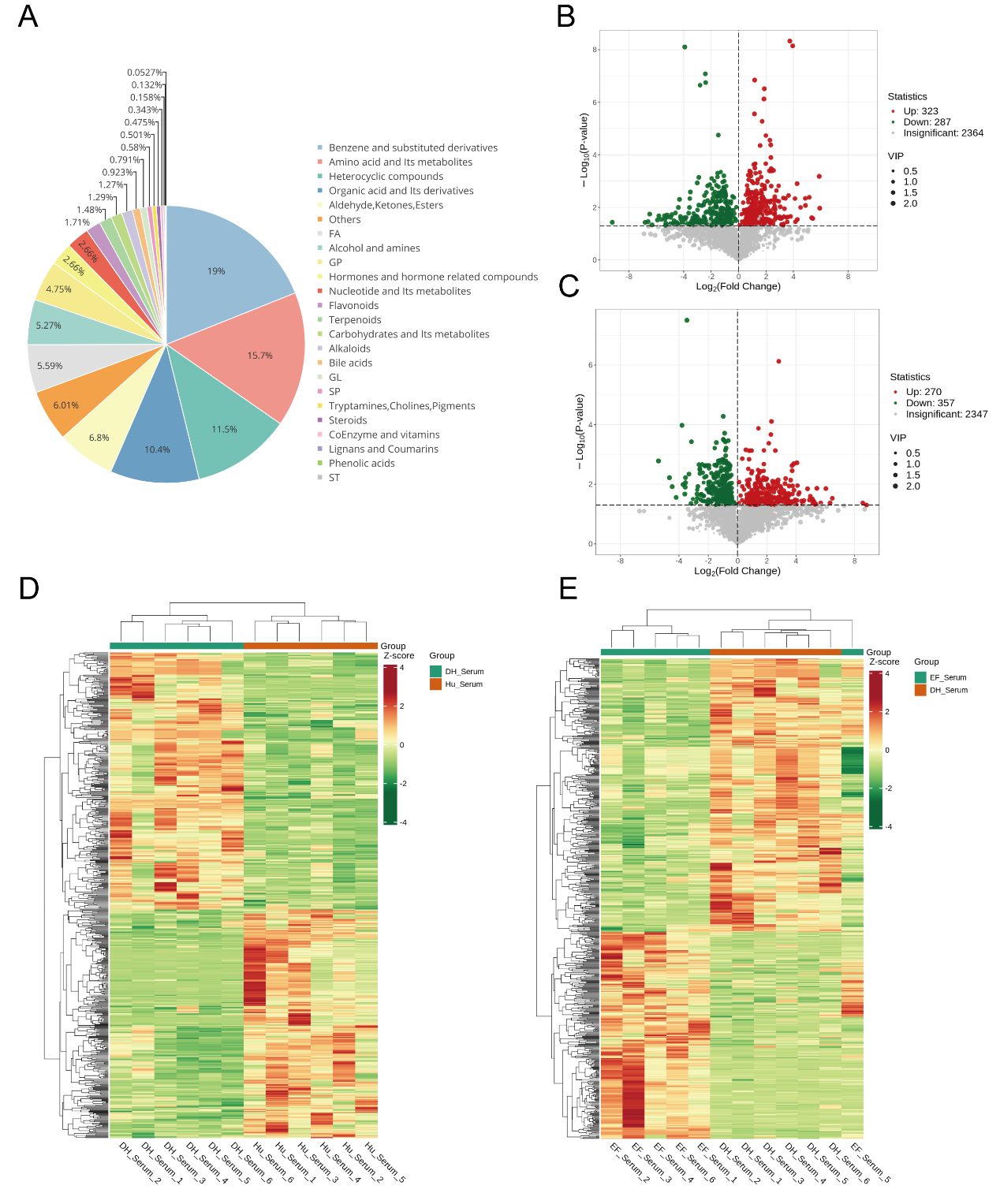


**Figure S13** Spearman correlation analysis was performed to assess the relationship between immune indicators and the rumen microbiota in DH, Hu, and EF sheep. For each group, microbial taxa with LDA scores > 4 identified by LEfSe analysis were correlated with the respective immune parameters. Panels (A), (B), and (C) represent the correlation results for DH, Hu, and EF sheep, respectively.


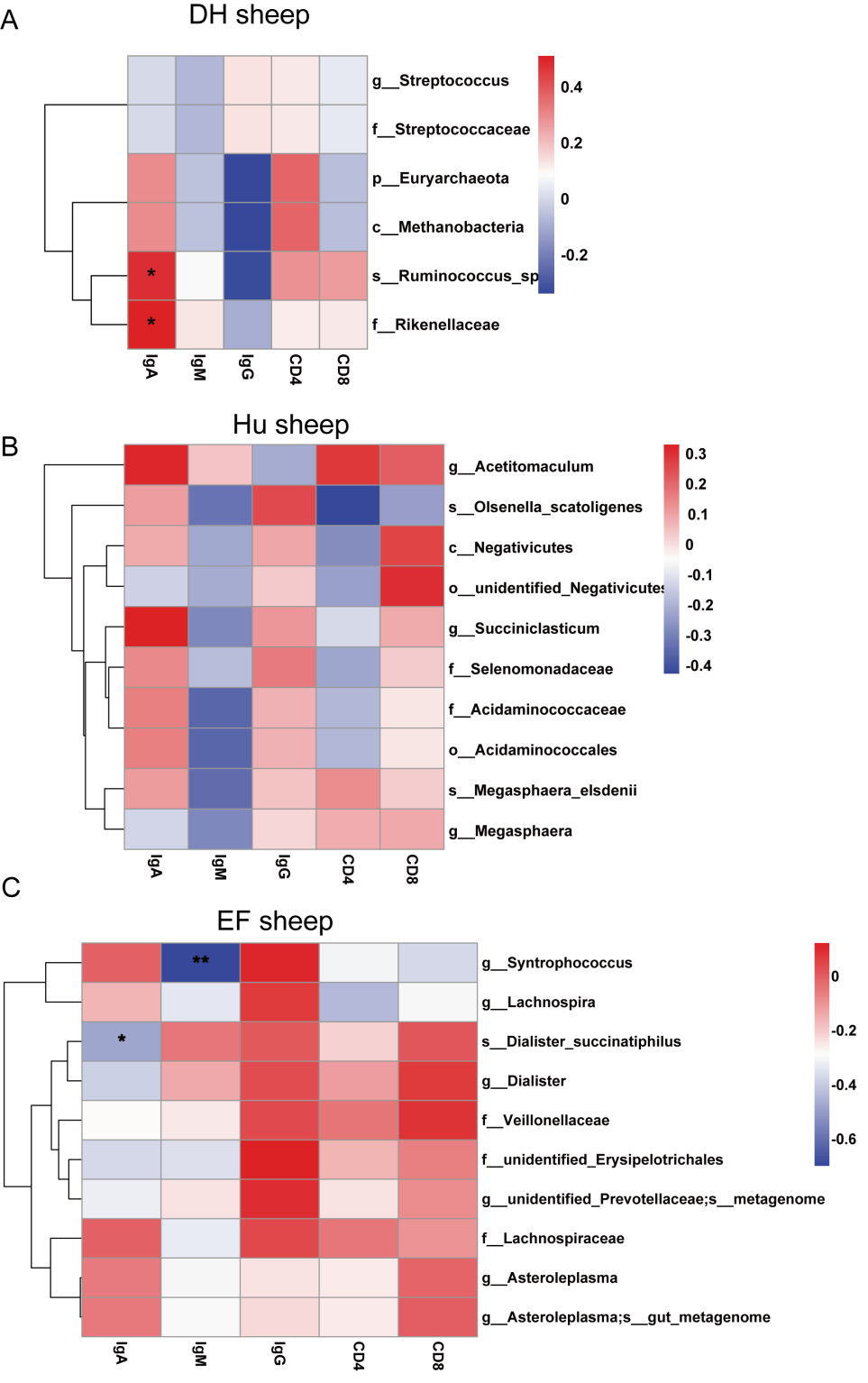

Supplement: Supplementary file 1 [file Data_Sheet_1.docx]
